# Limitations of Molecular Docking in Predicting the Selectivity of Selective Androgen Receptor Modulators (SARMs): A Comparative Study of YK11 and Ostarine Across Five Nuclear Receptors

**DOI:** 10.3390/ijms27135765

**Published:** 2026-06-26

**Authors:** Kaloyan Mihalev, Ivelin Iliev, Nadya Agova, Nikolay Toshev, Svetlana Georgieva

**Affiliations:** 1 Department of Pharmaceutical Chemistry, Faculty of Pharmacy, Medical University of Varna, 9000 Varna, Bulgaria; 2 Department of Bioorganic Chemistry, Faculty of Pharmacy, Medical University of Plovdiv, 4002 Plovdiv, Bulgaria

**Keywords:** selective androgen receptor modulators, YK11, ostarine, molecular docking, androgen receptor, steroid hormone receptors, nuclear receptor cross-reactivity, ligand–receptor interactions, computational pharmacology

## Abstract

Selective androgen receptor modulators (SARMs) are commonly described as tissue-selective anabolic agents, yet the extent to which this selectivity is reflected at the level of receptor-binding energetics remains uncertain. This study evaluated the receptor interaction profiles of the steroidal SARM YK11 and the nonsteroidal SARM ostarine across five steroid hormone nuclear receptors. Flexible molecular docking was performed with AutoDock 4.2 against the androgen (AR), estrogen (ER), progesterone (PR), glucocorticoid (GR), and mineralocorticoid (MR) receptors, using testosterone, estradiol, progesterone, cortisol, and aldosterone as endogenous reference ligands. Binding free energy, docking-derived inhibition constants, intermolecular interaction energies, conformational sampling, and two-dimensional interaction maps were analyzed. Ostarine showed favorable binding across all receptor systems, with binding energies ranging from −10.42 to −12.05 kcal/mol and no pronounced energetic preference for the androgen receptor. YK11 displayed stronger predicted binding, particularly toward the glucocorticoid, progesterone, and androgen receptors, with a docking energy trend of GR > PR > AR > MR > ER. Interaction analysis revealed conserved polar anchoring residues across receptor pockets, together with scaffold-specific contacts that may explain cross-receptor compatibility. These findings indicate that, within the AutoDock 4.2 flexible docking framework applied in this study, docking-derived binding energies primarily describe thermodynamic compatibility with nuclear receptor ligand-binding domains and should not be interpreted as direct predictors of functional SARM tissue selectivity. The observed discordance between predicted receptor affinity and the established tissue-selective pharmacology of ostarine highlights the need for caution when using single-method docking workflows to infer selectivity among closely related steroid hormone receptors. The novelty of this study lies in demonstrating, using a defined AutoDock 4.2-based comparative protocol, that receptor-binding energetics alone do not recapitulate the functional tissue-selective behavior attributed to SARMs.

## 1. Introduction

The androgen receptor (AR) is a ligand-activated member of the nuclear steroid hormone receptor superfamily that plays a central role in the regulation of skeletal muscle mass, bone homeostasis, reproductive physiology, metabolism, and aspects of cognition and behavior [1,2,3]. Upon binding endogenous androgens such as testosterone or dihydrotestosterone, the receptor undergoes conformational change, dissociates from cytosolic chaperones, translocates to the nucleus, and regulates the transcription of androgen-responsive genes [4,5]. Although this signaling axis is essential for normal physiology, its pharmacological manipulation with testosterone or anabolic-androgenic steroids is inherently limited by the broad distribution of AR across tissues and by the consequent overlap between desirable anabolic effects and undesirable androgenic effects [6]. In clinical practice, conventional androgen therapy may improve lean body mass, bone density, sexual symptoms, and quality of life in selected patients, yet its use remains constrained by concerns related to erythrocytosis, prostate stimulation, fertility suppression, lipid alterations, hepatotoxicity, and endocrine disruption [7,8,9,10,11].

Selective androgen receptor modulators (SARMs) emerged from the effort to dissociate anabolic actions from classical androgenic liabilities [12]. Conceptually analogous to selective estrogen receptor modulators, SARMs are small molecules capable of acting as full agonists, partial agonists, or functionally tissue-selective ligands of the AR, depending on their chemical structure and the cellular context in which receptor signaling occurs [13]. Their therapeutic appeal lies in the possibility of promoting anabolic activity in muscle and bone while reducing stimulation of androgen-sensitive tissues such as the prostate, skin, and hair follicles [14,15]. Although the exact molecular basis of tissue selectivity is not fully understood, it is widely attributed to ligand-specific conformational changes in the AR, differential recruitment of cofactors, and tissue-dependent transcriptional programs [16]. This mechanistic framework has made SARMs attractive candidates for disorders characterized by muscle wasting, frailty, hypogonadism, osteoporosis, cancer cachexia, benign prostatic hyperplasia, selected breast cancers, and other conditions in which androgen signaling may be beneficial but traditional androgen therapy is suboptimal [17].

Despite this promise, the translational history of SARMs has been mixed. Early pre-clinical and clinical work suggested that several compounds could improve lean body mass and functional measures while producing fewer overt androgenic effects than anabolic steroids [18,19,20]. Among the best-studied compounds, ostarine (also known as enobosarm or MK-2866) became one of the most extensively developed nonsteroidal SARMs and is often regarded as a prototype for the class [21]. It was advanced primarily in the setting of muscle wasting and cancer cachexia, where it showed the ability to increase lean body mass, improve stair-climbing performance, and reduce body fat in early-phase studies [22,23,24]. Ostarine also attracted attention because of favorable theoretical properties frequently ascribed to nonsteroidal SARMs, including oral bioavailability, lack of aromatization to estrogens, and reduced interaction with 5α-reductase-dependent pathways that contribute to dihydrotestosterone-mediated adverse effects [25,26,27]. At the same time, these encouraging signals did not translate into straightforward clinical adoption, and broader reviews have emphasized that, despite decades of investigation, SARM agonists have not secured the kind of routine therapeutic role that was once anticipated [28].

Ostarine remains particularly important in the present context because it sits at the intersection of therapeutic development and real-world misuse [29]. From a biomedical standpoint, it represents one of the clearest examples of how the SARM concept was intended to work: meaningful anabolic benefit with attenuated androgenic burden. From a practical standpoint, however, ostarine also became one of the most visible SARMs in recreational use, bodybuilding communities, and anti-doping casework [30,31,32]. This dual identity makes it a useful comparator when evaluating other members of the class, especially compounds that are marketed more aggressively, less clinically characterized, or structurally closer to anabolic steroids. In studies and reviews addressing misuse and adverse events, ostarine is repeatedly identified among the most common SARMs encountered in supplements, online products, and doping-related settings, highlighting its relevance not only as a pharmacological lead but also as a public health and sports-integrity concern [33].

Within this landscape, YK11 occupies a distinct and particularly controversial position. Unlike archetypal nonsteroidal SARMs such as ostarine, YK11 is a steroidal AR ligand with a chemical scaffold much closer to anabolic steroids. This molecule has been described in the literature as a partial androgen receptor agonist with gene-selective properties, based on in vitro functional assays, including AR-dependent transcriptional activity and follistatin upregulation [34]. Much of the interest surrounding it derives from its reported ability to increase follistatin expression and thereby functionally suppress myostatin signaling. Because myostatin is a negative regulator of skeletal muscle growth, the YK11-follistatin-myostatin axis has been widely invoked to explain the compound’s reputation as an unusually potent anabolic agent [35]. Experimental studies cited in the literature suggest that YK11 may regulate myogenic differentiation and promote osteoblastic proliferation and differentiation, thereby reinforcing its image as a compound of potential relevance for muscle and bone biology [36]. At the same time, the available evidence base for YK11 is remarkably limited compared with the scale of attention it has received in fitness and enhancement-oriented communities.

The attraction of YK11 is therefore driven not by mature clinical evidence, but by a convergence of pharmacological speculation, high anabolic expectations, and market mythology. Recent work has extended the discussion of YK11 beyond muscle growth and suggested that its biological profile may be more complex, and potentially more hazardous, than often assumed [37]. In rodent experiments, YK11 administration has been associated with oxidative stress, impairment of antioxidant defenses, mitochondrial dysfunction, and neurochemical toxicity in the hippocampus, raising concerns that the compound may not simply represent a “safer anabolic alternative” [38]. Moreover, because YK11 bears greater structural resemblance to steroidal androgenic agents than many non-steroidal SARMs, it has been hypothesized that it may retain a broader capacity for interaction with steroid hormone receptor systems or for producing off-target endocrine and neurochemical effects [39]. These considerations are highly relevant when discussing selectivity, because the common narrative around SARMs often equates anabolic selectivity with receptor exclusivity, whereas actual nuclear receptor cross-reactivity may be considerably more complex.

The gap between the theoretical promise of SARMs and their real-world use is further amplified by the rapidly expanding nonmedical market for these agents [40]. Recent reviews show that SARMs are widely promoted as performance-enhancing substances, often sold online, frequently mislabeled, and commonly consumed outside any clinical supervision [41]. Their appeal is particularly strong among bodybuilders, recreational gym users, and competitive athletes seeking increases in muscle mass, reductions in fat mass, faster recovery, or an alternative to traditional anabolic steroids [42,43]. This pattern of use has important regulatory and toxicological implications. The U.S. FDA has repeatedly warned that SARMs are unapproved drugs, not lawful dietary supplements, and has linked them to serious adverse outcomes, including liver injury, cardiovascular risk, psychiatric effects, infertility, and other harms [44,45]. In parallel, WADA continues to classify SARMs as prohibited anabolic agents in sport, reflecting both their enhancement potential and their established role in doping control [46]. Recent clinical and case-based reviews additionally show that the adverse-event literature is increasingly dominated by reports of drug-induced liver injury, often in the setting of uncertain dose, unknown purity, or concomitant use of multiple performance-enhancing compounds [47,48,49,50,51].

These issues make receptor-level selectivity a question of more than purely theoretical interest. For compounds advertised as selective, the extent to which they truly discriminate between the androgen receptor and other closely related nuclear hormone receptors remains incompletely defined [52]. This is especially relevant for YK11, whose steroidal character may predispose it to broader receptor promiscuity, and for ostarine, which is often cited as a more canonical example of a selective androgen receptor modulator [53]. The structural homology shared by the androgen, progesterone, glucocorticoid, mineralocorticoid, and estrogen receptor systems raises the possibility that compounds optimized for AR signaling may still exhibit meaningful affinity toward other steroid receptor binding pockets [54]. Such cross-reactivity could help explain discordance between marketed claims of selectivity and the broader spectrum of biological effects, adverse events, or endocrine disturbances described in experimental and real-world settings [55]. It also provides a strong rationale for computational studies aimed at comparing binding tendencies across multiple steroid hormone receptors, rather than restricting the analysis to AR alone.

The present study was designed to examine the receptor interaction profile of YK11 in comparison with ostarine using molecular docking across a panel of steroid hormone receptors. By placing a highly marketed, mechanistically intriguing, but poorly characterized steroidal SARM beside a clinically better characterized nonsteroidal comparator, the study addresses a central question in contemporary computational pharmacology: whether docking-derived receptor-binding energetics can adequately explain the functional selectivity attributed to SARMs, or whether these compounds may instead show broader binding compatibility across structurally related nuclear receptor systems. This analysis is relevant not only for pharmacological interpretation but also for toxicological risk assessment, rational drug design, and the broader discussion of the limitations of using molecular docking alone to infer selectivity among steroid hormone receptors.

In the present manuscript, the term receptor selectivity refers to the preferential predicted binding of a ligand to one steroid hormone receptor ligand-binding domain over another, as inferred from docking-derived binding free-energy and calculated inhibition constant values. In contrast, tissue selectivity refers to differential functional outcomes across tissues, such as anabolic effects in muscle or bone with reduced stimulation of androgen-sensitive tissues such as the prostate. These two concepts are not interchangeable. Docking can only address receptor-pocket compatibility and predicted receptor-binding preference, whereas tissue selectivity depends on post-binding events, including receptor conformation, coactivator/corepressor recruitment, transcriptional context, and tissue-specific signaling. Therefore, unless explicitly stated otherwise, the docking results in this study are interpreted as indicators of predicted receptor-binding preference rather than direct evidence of functional tissue selectivity.

The study was intentionally designed as a focused two-compound comparative analysis rather than as an exhaustive scaffold-wide evaluation of the SARM chemical space. Ostarine was selected as a representative nonsteroidal SARM because it is among the most extensively investigated compounds in this class and has been widely used as a reference compound in discussions of SARM pharmacology, clinical development, and misuse. In contrast, YK11 was selected as a structurally distinct steroidal SARM-like ligand because of its increasing visibility, limited experimental characterization, reported partial androgen receptor agonism, and growing attention in nonmedical and performance-enhancement contexts. Thus, the purpose of the study was not to infer universal conclusions for all SARMs, but to compare two pharmacologically and structurally contrasting examples in order to examine whether receptor-binding energetics generated by a defined AutoDock 4.2-based docking workflow could reproduce the functional selectivity attributed to these compounds.

## 2. Results

To enable a consistent and comparative evaluation of ligand-receptor interactions across the studied nuclear receptors, several docking parameters were analyzed, including binding free energy (ΔG, kcal/mol), inhibition constant (Ki), intermolecular interaction energy (E_inter_), torsional free energy (E_tors_), total internal energy, and selected cluster rank and selected cluster population. These parameters collectively describe both the thermodynamic favorability of ligand binding and the structural adaptability of the ligand within the receptor binding pocket.

The primary criterion for ranking docking results was the calculated AutoDock 4.2 binding energy (ΔG), where more negative values indicate a more stable and energetically favorable ligand–receptor complex. Correspondingly, the inhibition constant (Ki), derived from ΔG, provides an estimate of binding strength in concentration terms; lower Ki values reflect higher binding affinity and stronger interaction between ligand and receptor. In general, ligands with subnanomolar or low nanomolar Ki values are considered to exhibit more favorable docking-derived interaction estimates under the applied in silico conditions.

Because the reported docking values represent best-ranked poses from stochastic docking runs, differences in predicted binding energy were interpreted as method-dependent ranking trends rather than statistically validated differences in receptor binding or selectivity.

Throughout the manuscript, the term predicted binding affinity is used to refer to docking-derived estimates based on calculated binding free energy and the corresponding inhibition constant. These values should not be interpreted as experimentally measured affinity constants.

The intermolecular interaction energy (E_inter_) reflects the strength of direct non-covalent interactions between the ligand and the receptor, including hydrogen bonding, van der Waals forces, hydrophobic interactions, and π–π contacts. More negative values indicate stronger stabilizing interactions within the binding pocket. In contrast, E_tors_ represents the energetic penalty associated with ligand flexibility and conformational adaptation during binding; higher values suggest greater internal strain, which may reduce the overall stability of the complex.

The total internal energy provides additional insight into the intrinsic stability of the ligand conformation, while the unbound energy reflects the reference energetic state of the ligand prior to binding. Although these parameters contribute to the overall energy balance, they are primarily considered supportive descriptors rather than primary indicators of docking-derived binding behavior.

The selected cluster population provides information on pose convergence and the representativeness of the reported lowest-energy conformation. A higher selected cluster population indicates that a larger proportion of independent docking runs converged toward the same binding mode, whereas a low population suggests more dispersed docking behavior.

RMSD values were not used as comparative indicators of docking quality in the main results because the investigated ligands differ substantially in scaffold, size, and conformational flexibility. Therefore, the comparative interpretation focused on binding free energy, docking-derived inhibition constant, intermolecular energy, torsional contribution, and interaction fingerprints.

The comparative analysis in this study focuses primarily on ΔG and docking-derived Ki as the main scoring-based descriptors of ligand-receptor interaction, supported by E_inter_ and E_tors_ for interpretation of interaction strength and conformational behavior. The combination of these parameters enables a comprehensive assessment of ligand-binding profiles and facilitates the evaluation of receptor selectivity across the investigated nuclear receptors. The complete AutoDock 4.2 docking output values for all receptor–ligand combinations, including conformations, RMSD, binding energy, docking-derived Ki, intermolecular energy, total internal energy, torsional free energy, and unbound energy, are provided in Supplementary Table S2.

### 2.1. Androgen Receptor Binding (PDB ID: 2AM9)

The molecular docking analysis revealed distinct differences in the docking-derived energetic profiles of the three investigated ligands toward the androgen receptor (PDB ID: 2AM9). The results, expressed as binding free energy (ΔG, kcal/mol) and the corresponding inhibition constant (Ki), are summarized in Table 1. YK11 exhibited the lowest binding free energy (ΔG = −13.57 kcal/mol), indicating the formation of the most energetically favorable complex with the androgen receptor. This value is slightly more favorable than that of the endogenous ligand testosterone (ΔG = −13.42 kcal/mol), suggesting a comparable, and potentially more favorable, docking-derived binding energy. In contrast, ostarine showed a less favorable docking-derived binding energy (ΔG = −11.08 kcal/mol), corresponding to a markedly weaker interaction with the receptor. These findings are further supported by the calculated inhibition constants. YK11 and testosterone showed subnanomolar binding affinity, with Ki values of 113.14 pM and 145.77 pM, respectively, whereas ostarine exhibited a Ki in the nanomolar range (7.52 nM), indicating approximately two orders of magnitude lower binding affinity compared to the other two ligands.

Analysis of E_inter_ confirms this trend. YK11 demonstrated the most favorable intermolecular interaction energy (−14.76 kcal/mol), reflecting strong non-covalent interactions within the binding pocket. Testosterone also showed favorable interactions (−13.42 kcal/mol), while ostarine displayed weaker intermolecular stabilization (−12.87 kcal/mol). These differences suggest that the binding of YK11 is driven by a more optimal complementarity with the receptor’s active site.

The E_tors_ were minimal for all ligands, although ostarine exhibited the highest torsional penalty (+1.79 kcal/mol), consistent with its greater conformational flexibility. In contrast, testosterone, as a rigid steroidal molecule, showed no torsional penalty, which contributes to the stability of its binding. YK11 displayed an intermediate torsional contribution (+1.19 kcal/mol), indicating a balance between structural rigidity and adaptability within the binding site.

Cluster analysis further supported the representativeness of the selected docking poses. For all three ligands, the lowest-energy conformation belonged to cluster rank 1. The selected cluster population was highest for testosterone (88/100 runs), followed by YK11 (52/100 runs), indicating strong convergence toward the reported binding modes. In contrast, ostarine showed a much lower selected cluster population (2/100 runs), suggesting limited clustering convergence for this ligand within the androgen receptor binding pocket. Therefore, although the ostarine pose was selected according to the lowest binding free energy criterion, its lower cluster population supports a more cautious interpretation of its docking orientation compared with YK11 and testosterone.

### 2.2. Estrogen Receptor Binding (PDB ID: 1A52)

The docking results obtained for the estrogen receptor (PDB ID: 1A52) indicate clear differences in the interaction profiles of YK11, ostarine, and the endogenous ligand estradiol, as shown in Table 2. Among the three ligands, YK11 produced the most favorable binding free energy (ΔG = −11.32 kcal/mol), pointing to the most favorable docking-derived energetic profile toward the receptor. Its binding energy was slightly lower than that of estradiol (ΔG = −10.88 kcal/mol), whereas ostarine showed the least favorable value (ΔG = −10.42 kcal/mol), although still compatible with stable binding within the receptor cavity.

This ranking was mirrored by the calculated inhibition constants. YK11 yielded the lowest Ki value (5.02 nM), followed by estradiol (10.67 nM), while ostarine displayed the highest Ki (22.99 nM). Taken together, these data suggest that, under the applied docking conditions, YK11 has the strongest predicted interaction with the estrogen receptor, whereas ostarine shows the weakest of the three ligands.

The E_inter_ values calculated for YK11 (−12.52 kcal/mol) and ostarine (−12.21 kcal/mol) were both more favorable than that of estradiol (−10.88 kcal/mol), indicating stronger direct non-covalent contacts with the receptor environment. This may reflect a greater contribution of hydrogen bonding, hydrophobic interactions, and van der Waals contacts in the case of the two synthetic ligands. Differences in torsional free energy also provide useful context for interpreting these results. Ostarine showed the highest torsional penalty (+1.79 kcal/mol), consistent with a greater energetic cost associated with conformational adaptation during binding. YK11 exhibited a smaller but still appreciable torsional contribution (+1.19 kcal/mol), whereas estradiol showed no torsional penalty (+0.00 kcal/mol), in agreement with its more rigid steroidal framework.

Cluster analysis showed that the selected lowest-energy conformations of all three ligands belonged to cluster rank 1. Estradiol displayed the highest selected cluster population (59/100 runs), indicating good convergence toward the reported binding mode. In contrast, YK11 and ostarine showed lower selected cluster populations, with 7/100 and 4/100 runs, respectively. This suggests more dispersed docking solutions for the two synthetic ligands within the estrogen receptor binding pocket. Therefore, although the reported YK11 and ostarine poses correspond to the lowest-energy clusters, their lower cluster populations indicate reduced pose convergence compared with estradiol.

### 2.3. Progesterone Receptor Binding (PDB ID: 1A28)

YK11 showed the most favorable binding free energy (ΔG = −14.03 kcal/mol), corresponding to the most stable predicted complex in this receptor system (Table 3). Its ΔG value was lower than that of the endogenous ligand progesterone (−13.04 kcal/mol), whereas ostarine produced the least favorable result (−12.05 kcal/mol), despite remaining within a range compatible with stable receptor binding.

The same tendency is reflected in the calculated inhibition constants. YK11 yielded the lowest Ki value (51.93 pM), indicating the most favorable docking-derived estimate for the progesterone receptor. Progesterone also displayed very strong binding, with a Ki of 277.38 pM, while ostarine was associated with a substantially higher Ki (1.46 nM). Under these docking conditions, the binding affinity of YK11 therefore appears stronger than that of the endogenous ligand and markedly higher than that predicted for ostarine.

The most favorable E_inter_ value was again observed for YK11 (−15.22 kcal/mol), suggesting particularly efficient non-covalent stabilization within the receptor binding pocket. Ostarine and progesterone showed less favorable intermolecular energies (−13.84 and −13.34 kcal/mol, respectively), consistent with weaker overall stabilization of their docked complexes. Ostarine displayed the highest torsional penalty (+1.79 kcal/mol), in line with its greater conformational flexibility and the higher energetic cost of adopting a bound conformation. YK11 showed an intermediate torsional contribution (+1.19 kcal/mol), whereas progesterone exhibited only a minimal torsional penalty (+0.30 kcal/mol), which is consistent with the comparatively rigid nature of its steroidal scaffold.

Cluster analysis showed that the selected lowest-energy conformations of all three ligands belonged to cluster rank 1. The selected cluster population was highest for progesterone (52/100 runs), followed closely by YK11 (47/100 runs), indicating good convergence toward the reported binding modes for the two steroidal ligands. Ostarine showed a lower selected cluster population (8/100 runs), suggesting a more dispersed docking behavior within the progesterone receptor binding pocket. Thus, the YK11 and progesterone docking poses can be considered well represented by their respective clusters.

### 2.4. Glucocorticoid Receptor Binding (PDB ID: 4P6X)

As presented in Table 4, cortisol produced the most favorable binding free energy (ΔG = −15.14 kcal/mol), consistent with the most stable predicted complex in this receptor system. YK11 followed closely, with a ΔG of −14.79 kcal/mol, indicating a comparably strong interaction with the glucocorticoid receptor. By contrast, ostarine displayed a clearly weaker binding profile, with a binding energy of −11.60 kcal/mol.

Cortisol showed the lowest Ki value (7.96 pM), as expected for the physiological ligand of the receptor. YK11 likewise remained in the picomolar range (14.32 pM), pointing to very high predicted binding affinity, whereas ostarine exhibited a much higher Ki (3.12 nM), indicative of substantially weaker receptor binding. Although YK11 did not exceed cortisol in overall binding affinity, its binding remained notably close to that of the endogenous ligand and far stronger than that predicted for ostarine.

A slightly different perspective emerges from the intermolecular interaction energy values. Here, YK11 showed the most favorable E_inter_ (−15.99 kcal/mol), marginally surpassing cortisol (−15.44 kcal/mol), while ostarine again ranked lowest (−13.39 kcal/mol). This suggests that YK11 is capable of establishing particularly strong direct non-covalent interactions within the receptor pocket, even though the overall binding free energy remains slightly less favorable than that of cortisol.

The torsional free energy term further distinguishes the three ligands. Ostarine showed the highest torsional penalty (+1.79 kcal/mol), consistent with a greater conformational cost upon binding. YK11 occupied an intermediate position (+1.19 kcal/mol), whereas cortisol displayed only a minimal torsional contribution (+0.30 kcal/mol), in line with the more rigid character of its steroidal structure.

Cluster analysis showed that the selected lowest-energy conformations of all three ligands belonged to cluster rank 1. YK11 and cortisol displayed selected cluster populations of 47/100 and 39/100 runs, respectively, indicating good convergence toward the reported binding modes. In contrast, ostarine showed a selected cluster population of only 1/100 runs, indicating that the lowest-energy pose was not supported by a broadly populated docking cluster. Therefore, although the ostarine conformation was retained according to the lowest binding free energy criterion, its exact orientation within the glucocorticoid receptor binding pocket should be interpreted with particular caution. This observation is consistent with the reduced conformational convergence already suggested by the limited number of ostarine docking poses in this receptor system.

### 2.5. Mineralocorticoid Receptor Binding (PDB ID: 2AA2)

Docking to the mineralocorticoid receptor (PDB ID: 2AA2) showed discernible differences among YK11, ostarine, and the endogenous ligand aldosterone in terms of both binding affinity and energetic profile (Table 5). As expected for the physiological ligand of this receptor, aldosterone yielded the most favorable binding free energy (ΔG = −13.55 kcal/mol). YK11 followed with a less favorable but still clearly negative ΔG value of −12.28 kcal/mol, consistent with stable binding within the receptor cavity. Ostarine produced the least favorable result (ΔG = −11.57 kcal/mol), although its interaction remained energetically acceptable under the applied docking conditions.

The inhibition constants were fully consistent with this ranking. Aldosterone showed the lowest Ki value (117.20 pM), indicating the most favorable docking-derived estimate for the mineralocorticoid receptor. YK11 remained within the subnanomolar range, with a Ki of 989.11 pM, but its docking-derived energetic profile was clearly less favorable than that of the endogenous ligand. Ostarine displayed the highest Ki value (3.31 nM), in line with the weakest predicted binding among the three compounds.

In contrast to the more pronounced differences observed for ΔG and Ki, the intermolecular interaction energies were relatively close in magnitude. Aldosterone again showed the most favorable E_inter_ value (−13.85 kcal/mol), while YK11 (−13.48 kcal/mol) and ostarine (−13.36 kcal/mol) followed closely. This suggests that all three ligands are capable of establishing favorable direct non-covalent contacts within the mineralocorticoid receptor binding pocket, whereas the observed differences in overall binding affinity likely arise from the contribution of additional energetic terms. Ostarine had the highest torsional penalty (+1.79 kcal/mol), indicating the greatest conformational cost associated with binding. YK11 showed an intermediate torsional contribution (+1.19 kcal/mol), whereas aldosterone exhibited only a minimal torsional penalty (+0.30 kcal/mol), consistent with the rigid steroidal architecture of the endogenous ligand.

Cluster analysis showed that the selected lowest-energy conformations of all three ligands belonged to cluster rank 1. The selected cluster population was highest for YK11 (69/100 runs), followed closely by aldosterone (66/100 runs), indicating strong convergence toward the reported binding modes. Ostarine showed a lower selected cluster population (9/100 runs), suggesting more limited convergence compared with the steroidal ligands. Thus, the YK11 and aldosterone poses can be considered well represented by their respective docking clusters, whereas the exact orientation of ostarine should be interpreted more cautiously despite its assignment to the lowest-energy cluster.

### 2.6. Molecular Interaction Analysis of YK11 with Nuclear Receptor Ligand-Binding Domains

The interaction profiles of YK11 with the ligand-binding domains of all five investigated nuclear receptors were analyzed using the Discovery Studio Visualizer (v21.1.0.20298; Dassault Systèmes, San Diego, CA, USA). For each receptor, flexible docking was performed with selected active-site residues defined as rotatable, and the resulting binding poses were analyzed to identify the type and identity of intermolecular interactions between the ligand and the surrounding amino acid residues.

**Figure 1:**
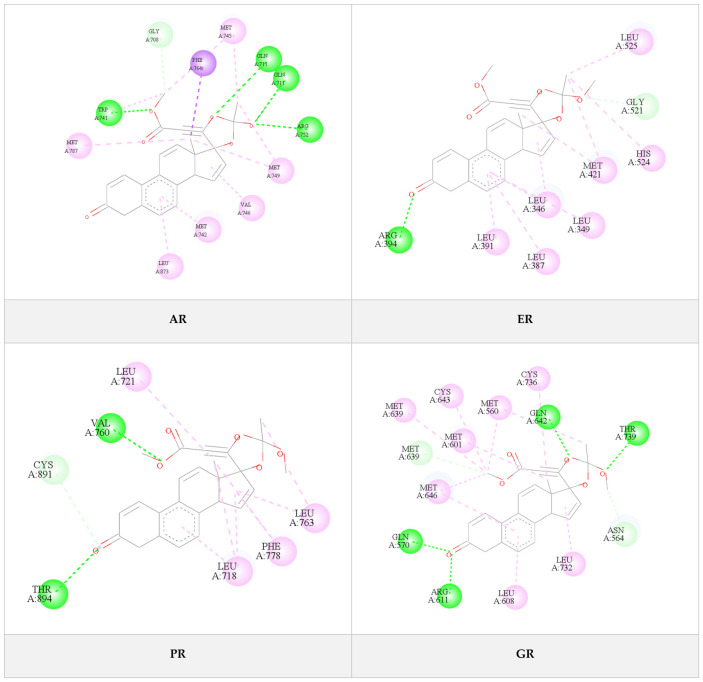

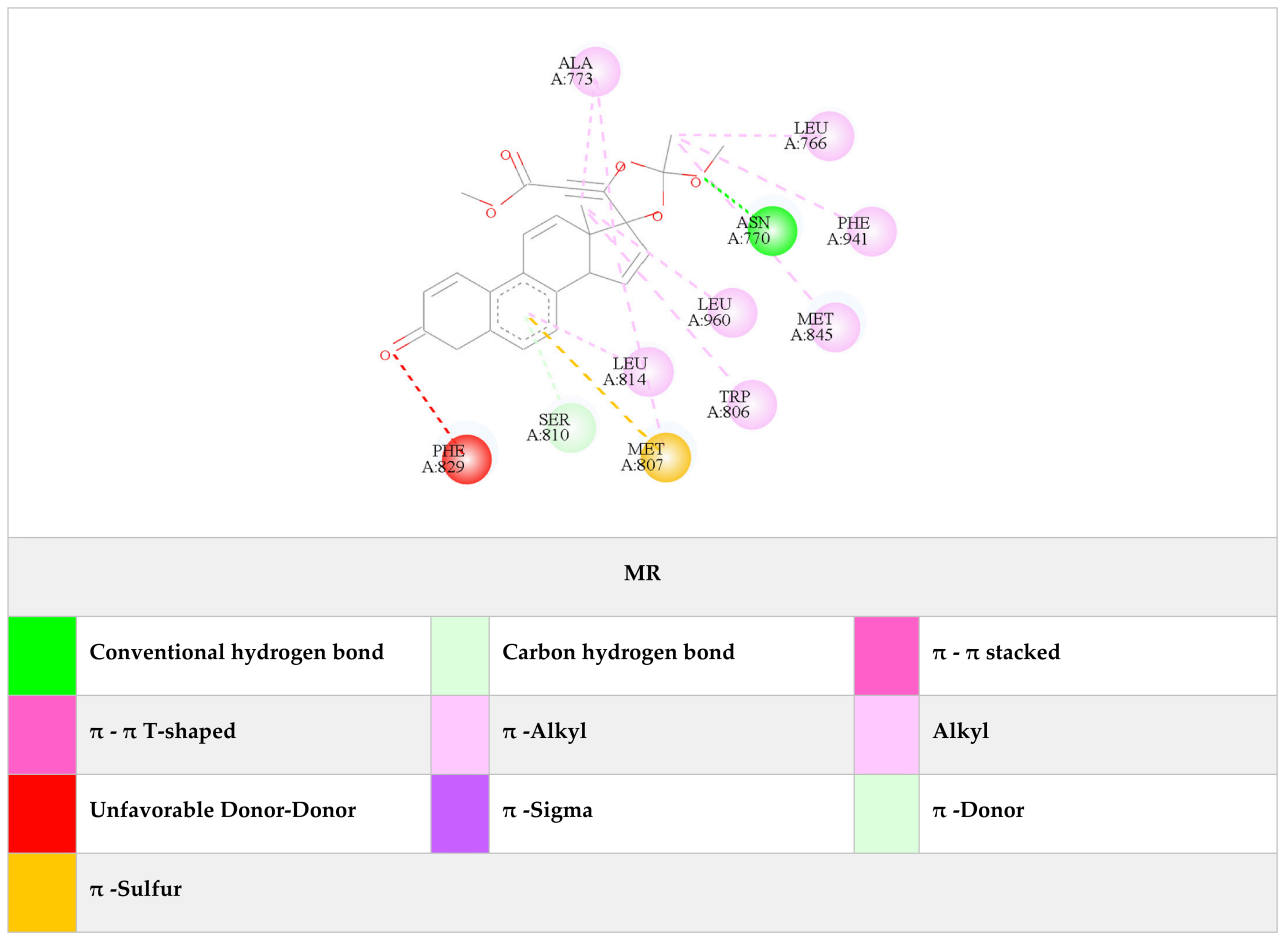
Two-dimensional interaction maps of YK11 within the ligand-binding domains of nuclear receptors: androgen (AR), estrogen (ER), progesterone (PR), glucocorticoid (GR), and mineralocorticoid (MR) receptors.

Within the androgen receptor ligand-binding domain, YK11 forms three conventional hydrogen bonds with Gln711, Trp741, and Arg752, as shown in Figure 1. The carbonyl oxygen of the ester group at C21 and the dioxolane oxygens at C17 serve as the principal hydrogen-bond acceptors. A carbon–hydrogen bond is additionally observed with Gly708. The aromatic system of the D-ring fused to the butenolide moiety participates in a π–Sigma interaction with Phe764. The steroidal tetracyclic scaffold is further stabilized by an extensive network of alkyl and π–Alkyl contacts with Met742, Met745, Met749, Met787, Val746, and Leu873, which form the hydrophobic lining of the binding cavity.

Within the estrogen receptor ligand-binding domain, YK11 establishes one conventional hydrogen bond between the C3 carbonyl oxygen of the A-ring and Arg394. A carbon–hydrogen bond is formed with Gly521. The hydrophobic pocket accommodates the steroidal scaffold through alkyl and π–Alkyl interactions with Leu346, Leu349, Leu387, Leu391, Met421, Leu525, and His524. No π–Sigma or π–Sulfur interactions are observed for this receptor.

Within the progesterone receptor ligand-binding domain, YK11 forms two conventional hydrogen bonds: one between the C3 carbonyl oxygen of the A-ring and Thr894, and one between the ester carbonyl oxygen of the C21 substituent and Val760. A carbon–hydrogen bond is observed with Cys891. Hydrophobic stabilization is provided by alkyl and π–Alkyl interactions with Leu718, Leu721, Leu763, and Phe778. Of the five receptors examined, the PR binding pocket yields the richest hydrogen-bonding pattern alongside AR, with two strong conventional hydrogen bonds involving both major polar functional groups of the ligand.

Within the glucocorticoid receptor ligand-binding domain, YK11 forms the largest number of conventional hydrogen bonds among all five receptors. Four conventional hydrogen bonds are identified: the C3 carbonyl oxygen of the A-ring interacts with Gln570 and Arg611; the dioxolane oxygen at C17 interacts with Gln642; and the ester oxygen of the C21 group interacts with Thr739. Carbon–hydrogen bonds are additionally observed with Met639 and Asn564. The hydrophobic environment of the binding cavity is defined by alkyl and π–Alkyl contacts with Met560, Met601, Met646, Cys643, Cys736, Leu608, and Leu732. The richness of both polar and hydrophobic contacts in the GR binding pocket is consistent with the most favorable binding free energy observed for YK11 across the receptor panel (ΔG = −14.79 kcal/mol).

Within the mineralocorticoid receptor ligand-binding domain, YK11 forms one conventional hydrogen bond between the dioxolane oxygen at C17 and Asn770. A π–Donor hydrogen bond is observed between the aromatic system of YK11 and Ser810. A π–Sulfur interaction is identified between the aromatic ring and Met807. The hydrophobic contacts are provided by alkyl and π–Alkyl interactions with Leu766, Leu814, Leu960, Met845, Trp806, Phe941, and Ala773. Notably, an unfavorable acceptor–acceptor interaction is detected between the C3 carbonyl oxygen of the A-ring and Phe829, representing the only destabilizing contact observed for YK11 across the entire receptor panel. This clash, along with the reduced number of conventional hydrogen bonds compared to GR and PR, is consistent with the lower binding free energy of YK11 at MR (ΔG = −12.28 kcal/mol) relative to its binding affinity for GR (−14.79 kcal/mol) and PR (−14.03 kcal/mol).

### 2.7. Molecular Interaction Analysis of Ostarine with Nuclear Receptor Ligand-Binding Domains

**Figure 2:**
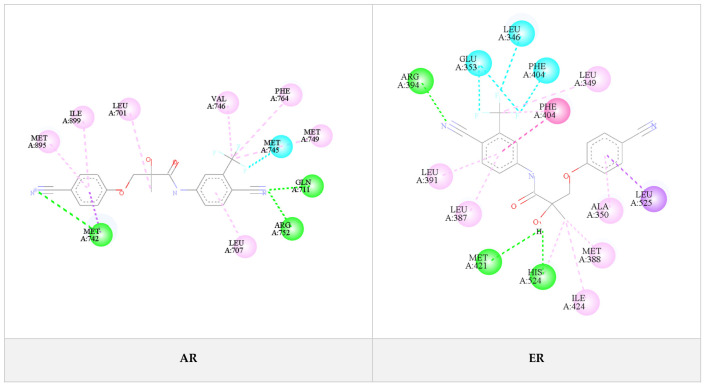

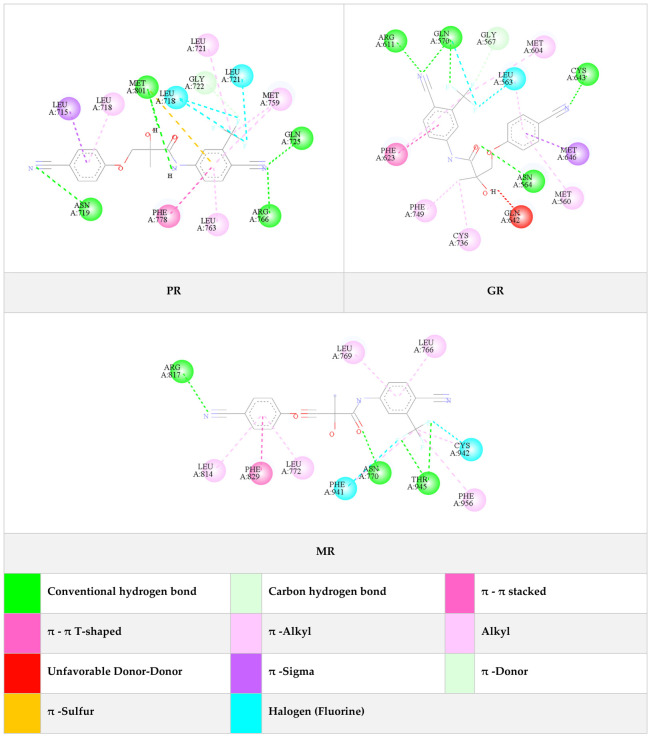
Two-dimensional interaction maps of ostarine within the ligand-binding domains of nuclear receptors: androgen (AR), estrogen (ER), progesterone (PR), glucocorticoid (GR), and mineralocorticoid (MR) receptors.

The interaction profiles of ostarine with the ligand-binding domains of all five investigated nuclear receptors were analyzed using Discovery Studio Visualizer, following flexible docking, and are shown in Figure 2. For each receptor, the type and identity of intermolecular interactions between the ligand and surrounding amino acid residues were recorded. Ostarine is a nonsteroidal arylpropionamide compound bearing two aromatic rings connected by a propanamide linker, a free hydroxyl group, a gem-dimethyl carbon, and a trifluoromethyl substituent. This scaffold differs substantially from the steroidal ligands and gives rise to a distinct interaction pattern across the receptor panel, including halogen bonds involving the three fluorine atoms, which are absent in the interaction profiles of YK11.

Within the androgen receptor ligand-binding domain, ostarine forms two conventional hydrogen bonds: the cyano group of the cyanophenyl ring interacts with Gln711, and the nitrogen of the amide group interacts with Arg752. A halogen (fluorine) bond is observed between one of the trifluoromethyl fluorine atoms and Met745. The aromatic ring bearing the trifluoromethyl and cyano substituents participates in a π–Sigma interaction with Met742. The hydrophobic environment of the binding cavity is defined by alkyl and π–Alkyl contacts with Leu701, Leu707, Val746, Met749, Phe764, Met895, and Ile899.

Within the estrogen receptor ligand-binding domain, ostarine forms three conventional hydrogen bonds: the hydroxyl group of the propanamide linker interacts with both Met421 and His524, and the carbonyl oxygen of the amide group also participates in hydrogen bonding with Met421. A conventional hydrogen bond is additionally observed between the cyano group of the cyanophenoxy ring and Arg394. Halogen (fluorine) bonds are formed between the trifluoromethyl fluorine atoms and Leu346, Glu353, and Phe404. A π–π T-shaped aromatic interaction is observed between the trifluoromethylphenyl ring and Phe404. A π–Sigma interaction is identified between the cyanophenoxy ring and Leu525. Hydrophobic stabilization is provided by alkyl and π–Alkyl contacts with Leu349, Leu387, Leu391, Met388, Ala350, and Ile424. Of the five receptors, the ER yields the most complex interaction fingerprint for ostarine, with the largest number of distinct interaction types.

Within the progesterone receptor ligand-binding domain, ostarine forms three conventional hydrogen bonds: the cyano group of the cyanophenoxy ring interacts with Asn719; the amide NH interacts with Leu718 (acting as a hydrogen-bond acceptor via its backbone carbonyl); and the cyano group of the trifluoromethylcyanophenyl ring interacts with both Arg766 and Gln725. A carbon-hydrogen bond is observed with Gly722. Halogen (fluorine) bonds are formed between the trifluoromethyl group and Leu721 and Gly722. A π–Sulfur interaction is identified between the aromatic ring of ostarine and Met801. A π–π stacked interaction is observed between the cyanophenyl ring and Phe778. A π–Sigma interaction is present with Leu715. Hydrophobic contacts are provided by alkyl and π–Alkyl interactions with Leu718, Leu721, Leu763, and Met759. The PR binding pocket yields the richest and most diverse interaction profile for ostarine across all five receptors, encompassing seven distinct interaction types.

Within the glucocorticoid receptor ligand-binding domain, ostarine forms four conventional hydrogen bonds: the cyano group interacts with Arg611 and Gln570; the hydroxyl group interacts with Asn564; and a hydrogen bond is formed between the amide nitrogen and Cys643. A carbon-hydrogen bond is additionally observed with Gly567. A halogen (fluorine) bond is formed between one trifluoromethyl fluorine atom and Leu563. An unfavorable donor-donor interaction is detected between the hydroxyl group and Gln642, representing a destabilizing contact within the binding pocket. π–π T-shaped interactions are observed with Phe623 and Phe749. A π–Sigma interaction is identified between the cyanophenyl ring and Met646. Hydrophobic contacts involve alkyl and π–Alkyl interactions with Met560, Met604, and Cys736.

Within the mineralocorticoid receptor ligand-binding domain, ostarine forms three conventional hydrogen bonds: the amide carbonyl oxygen interacts with Asn770 and Thr945, and the cyano group of the cyanophenoxy ring interacts with Arg817. Halogen (fluorine) bonds are observed between the trifluoromethyl fluorine atoms and Phe941 and Cys942. A π–π T-shaped interaction is identified between the cyanophenoxy ring and Phe829. Hydrophobic stabilization is provided by alkyl and π–Alkyl contacts with Leu766, Leu769, Leu772, Leu814, and Phe956.

### 2.8. Molecular Interaction Analysis of Endogenous Ligands (Controls) with Nuclear Receptor Ligand-Binding Domains

**Figure 3:**
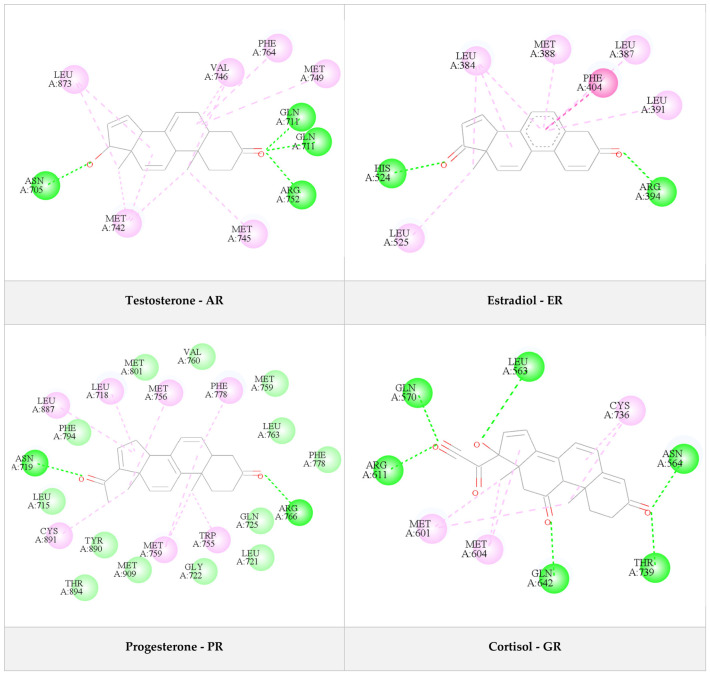

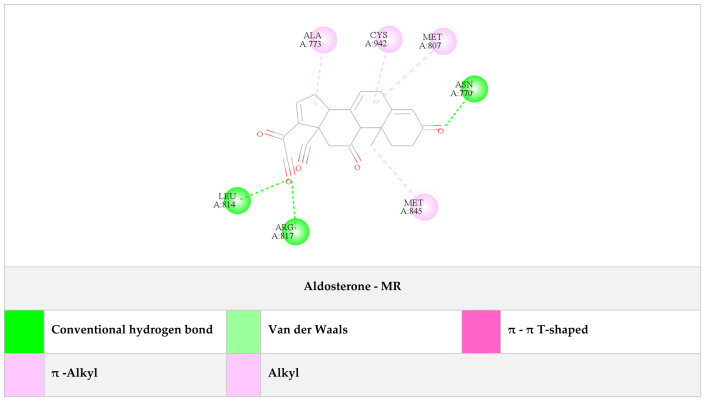
Two-dimensional interaction maps of endogenous ligands within the ligand-binding domains of their corresponding nuclear receptors (AR, ER, PR, GR, and MR).

To provide a reference framework for interpreting the binding behavior of YK11 and ostarine, the interaction profiles of the five endogenous ligands, testosterone (AR), estradiol (ER), progesterone (PR), cortisol (GR), and aldosterone (MR), were analyzed under identical docking conditions (Figure 3). All five controls are steroidal compounds and, as such, their interaction patterns are dominated by hydrophobic contacts arising from the complementarity of the rigid tetracyclic carbon framework with the hydrophobic ligand-binding domain cavities of the respective receptors.

Within the androgen receptor ligand-binding domain, testosterone forms three conventional hydrogen bonds: the C3 ketone oxygen interacts with Asn705, and the C17 hydroxyl group donates hydrogen bonds to Gln711 (two contacts resolved) and Arg752. No carbon–hydrogen bonds, π–Sigma interactions, or other polar contact types are observed. Hydrophobic stabilization is provided exclusively by alkyl and π–Alkyl interactions with Leu873, Met742, Val746, Met745, Met749, and Phe764, which form the nonpolar lining of the binding cavity.

Within the estrogen receptor ligand-binding domain, estradiol forms two conventional hydrogen bonds: the C17 hydroxyl group interacts with His524, and the C3 phenolic hydroxyl group interacts with Arg394. A π–π T-shaped aromatic interaction is observed between the A-ring of the steroidal scaffold and Phe404. Hydrophobic contacts are provided by alkyl and π–Alkyl interactions with Leu384, Leu387, Leu391, Met388, and Leu525.

Within the progesterone receptor ligand-binding domain, progesterone forms two conventional hydrogen bonds: the C3 ketone oxygen interacts with Asn719, and the C20 ketone oxygen interacts with Arg766. An extensive network of van der Waals contacts is additionally observed, involving Val760, Met801, Met759, Phe778, Leu763, Gln725, Leu721, Trp755, and Gly722. These van der Waals interactions arise from the close but non-covalent surface complementarity between the steroidal core and the surrounding residues and represent a distinctive feature of the progesterone-PR complex relative to the other four receptor systems examined. Hydrophobic alkyl and π–Alkyl contacts are formed with Leu718, Met756, Phe794, Leu715, Cys891, Tyr890, Met759, Met909, and Trp755. Of the five control ligand-receptor complexes, the progesterone-PR interaction profile is the most extensive, encompassing the largest number of residues and the unique presence of van der Waals interactions as a discrete interaction category.

Within the glucocorticoid receptor ligand-binding domain, cortisol forms six conventional hydrogen bonds, the largest number observed among all control ligands across the five receptor systems. The C3 ketone oxygen interacts with both Gln570 and Arg611; the C11 hydroxyl group interacts with Leu563; the C20 ketone oxygen interacts with Gln642; the C21 hydroxyl group interacts with Thr739; and an additional hydrogen bond is formed between a polar group of cortisol and Asn564. Hydrophobic stabilization is provided by alkyl contacts with Met601, Met604, and Cys736. No π–Alkyl or aromatic interactions are observed for this complex. The exceptionally rich hydrogen-bonding network of cortisol within the GR binding pocket is consistent with its highest binding free energy among all five endogenous ligands (ΔG = −15.14 kcal/mol).

Within the mineralocorticoid receptor ligand-binding domain, aldosterone forms three conventional hydrogen bonds: the C3 ketone oxygen interacts with Asn770; the C18 aldehyde oxygen forms hydrogen bonds with both Leu814 and Arg817. Hydrophobic stabilization is provided by alkyl contacts with Ala773, Cys942, Met807, and Met845. As with cortisol at the GR, no π–Alkyl or aromatic interactions are detected, and the interaction profile is defined entirely by conventional hydrogen bonds and alkyl contacts, reflecting the compact and geometrically precise binding mode of the endogenous mineralocorticoid.

To facilitate comparison of the qualitative interaction maps shown in Figure 1, Figure 2 and Figure 3, the interaction profiles were additionally summarized as ligand–receptor interaction fingerprints in Supplementary Table S3. This table provides a receptor-wise comparison of the main interaction categories observed in the best-ranked docking poses, including conventional hydrogen bonds, carbon–hydrogen bonds, halogen bonds, π–σ interactions, π–π interactions, π–alkyl/alkyl hydrophobic contacts, and unfavorable contacts where present. This summary allows direct comparison of interaction density and interaction-type diversity across the receptor panel. However, the interaction fingerprints should be interpreted as descriptive structural summaries rather than quantitative energetic decompositions, because individual hydrogen bond strengths, residue-wise energetic contributions, electrostatic surface potentials, and hydration effects were not calculated in the present AutoDock 4.2 workflow.

## 3. Discussion

The present study was designed to evaluate the receptor interaction profiles of two selective androgen receptor modulators—YK11 and ostarine (enobosarm, MK-2866), through flexible docking across a panel of five steroid hormone nuclear receptors: the androgen receptor (AR, PDB: 2AM9), estrogen receptor (ER, PDB: 1A52), progesterone receptor (PR, PDB: 1A28), glucocorticoid receptor (GR, PDB: 4P6X), and mineralocorticoid receptor (MR, PDB: 2AA2).

The central motivation for this analysis arises from a well-characterized discrepancy: ostarine is among the most extensively studied SARMs, with documented tissue-selective anabolic activity confirmed in preclinical and clinical settings, yet the docking data obtained in the present study do not reproduce this selectivity at the level of binding free energy. This discrepancy is not a methodological failure; rather, it constitutes the principal scientific finding of the study and raises a fundamental question about the capacity of molecular docking to capture the mechanistic basis of SARM tissue selectivity, even when a flexible docking protocol with defined rotatable residues within the active site is applied.

The selection of the receptor panel was based on both biological and pharmacological considerations. The androgen receptor was included as the primary pharmacological target of SARMs. The estrogen, progesterone, glucocorticoid, and mineralocorticoid receptors were included because they belong to the broader steroid hormone nuclear receptor family and contain structurally related ligand-binding domains capable of accommodating lipophilic steroidal or steroid-like ligands. This receptor panel is therefore relevant for evaluating whether compounds described as SARMs show predicted receptor-pocket compatibility beyond AR. Such a comparison is particularly important for YK11 because of its steroidal scaffold, which may increase the likelihood of compatibility with other steroid hormone receptor pockets, while ostarine provides a structurally distinct nonsteroidal comparator with established tissue-selective AR pharmacology. From an endocrine and oncology safety perspective, the inclusion of non-AR steroid hormone receptors is also relevant because unintended interaction with these receptors may contribute to off-target hormonal effects.

The tissue-selective pharmacology of ostarine has been established by a substantial body of experimental evidence that provides the essential reference point for interpreting the docking results. In vitro, ostarine binds the AR with high affinity (Ki ≈ 3.8 nM) and activates AR-dependent transcription in muscle cell models while producing markedly reduced stimulation of prostate cell proliferation relative to dihydrotestosterone (DHT) [56,57]. In vivo, oral administration in castrated rodent models increases levator ani muscle mass and bone mineral density at doses that only partially—and at low doses, minimally—increase prostate weight, demonstrating a favorable muscle-to-prostate anabolic:androgenic ratio that is a defining criterion of SARM pharmacology [58]. In phase 2 and phase 3 clinical trials (the POWER program), ostarine produced statistically significant increases in total lean body mass in cancer patients with muscle wasting, with an acceptable safety profile and no clinically significant androgenic adverse events [59]. This profile supports its classification as a tissue-selective AR modulator according to functional and clinical criteria, and this established biological reality must be held as the interpretive anchor against which the docking data are evaluated.

Against this well-established background, the docking analysis presented here reveals a pattern that superficially contradicts the concept of selectivity. Ostarine produced binding free energies ranging from −10.42 kcal/mol at ER to −12.05 kcal/mol at PR, with a value of −11.08 kcal/mol at AR, which is its primary pharmacological target. Across all five receptors, these energies correspond to inhibition constants in the low-to-mid nanomolar range (7.52 nM at AR, 22.99 nM at ER, 1.46 nM at PR, 3.12 nM at GR, and 3.31 nM at MR) without a pronounced energetic preference for AR over the other receptors. Viewed in isolation, this pattern would imply that ostarine interacts with all five receptors with comparable docking-derived energetic estimates, which is inconsistent with its documented in vivo selectivity. The resolution of this apparent contradiction lies not in questioning the experimental evidence for selectivity, but in understanding what docking energetics can and cannot measure.

YK11 exhibited a different but equally informative pattern. Its binding free energies ranged from −11.32 kcal/mol at ER to −14.79 kcal/mol at GR, with particularly favorable docking-derived energies also at PR (−14.03 kcal/mol) and AR (−13.57 kcal/mol). Strikingly, at AR, PR, and GR, YK11 matched or exceeded the calculated docking-derived energies of the respective endogenous ligands—testosterone (−13.42 kcal/mol), progesterone (−13.04 kcal/mol), and cortisol (−15.14 kcal/mol)—and the observed receptor-affinity hierarchy of GR > PR > AR > MR > ER is the inverse of what would be expected from a compound with genuine AR selectivity. The reliability of these results is supported by the docking validation performed with the five endogenous reference ligands, whose poses were consistently localized within the expected ligand-binding domains at energetically favorable values, confirming that the flexible docking protocol is capable of identifying the correct binding cavity for each receptor system. Unlike ostarine, YK11 lacks the extensive preclinical selectivity data that would allow a direct comparison between docking predictions and documented biological behavior. Its classification as a SARM-like compound is based on literature-reported in vitro functional evidence, including partial AR agonism, AR-dependent transcriptional responses, and follistatin-mediated modulation of myostatin-related signaling; however, these functional properties were not experimentally assessed in the present docking study, and validated in vivo receptor selectivity data equivalent to those available for ostarine do not exist.

### 3.1. Comparative Analysis of Ligand–Receptor Interaction Profiles

The interaction profiles obtained from flexible docking across the five receptor systems provide a mechanistic basis for interpreting the binding free energy hierarchy described above, and reveal three structurally distinct binding modes across the ligand panel that differ in interaction type, density, and spatial distribution.

In molecular docking studies, the interpretation of ligand–receptor recognition is commonly based not only on the docking score, but also on the spatial organization of hydrogen bonds, hydrophobic contacts, π-type interactions, halogen bonds, and unfavorable contacts within the binding site. Previous molecular interaction studies have shown that such non-covalent interaction patterns can help rationalize ligand accommodation, binding-site complementarity, and differences in predicted binding behavior, particularly when docking results are interpreted together with structural and physicochemical properties of the ligands [60,61]. Therefore, in the present study, the interaction maps were not interpreted as isolated descriptive outputs, but as structural fingerprints explaining how chemically distinct SARM scaffolds can satisfy conserved recognition features within related steroid hormone receptor pockets.

#### 3.1.1. Conserved Polar Anchoring Residues and the Structural Basis for Cross-Receptor Binding

A notable finding of the interaction analysis is the recurrence of a small set of polar residues that serve as hydrogen-bond partners for all three ligand types at each receptor, irrespective of scaffold. Within the AR binding pocket, Gln711 and Arg752 function as obligatory anchoring residues for all three AR ligands: testosterone contacts both via its ketone and hydroxyl oxygens, YK11 contacts them via the dioxolane and ester carbonyls of its C17 substituent, and ostarine engages the same residues via its cyano and amide nitrogen groups—three chemically distinct functional groups converging on the same two polar contacts. The same convergence is observed at the ER (Arg394 contacted by all three ER ligands via different chemical strategies), at the PR (Asn719 and Arg766 contacted by progesterone, YK11, and ostarine), at the GR (Gln570, Arg611, and Thr739 shared by cortisol, YK11, and ostarine), and at the MR (Asn770 and Arg817 contacted by all three MR ligands). The conservation of these polar anchor networks across ligands of vastly different molecular architecture provides the most direct structural explanation for why neither YK11 nor ostarine demonstrates clear AR selectivity at the level of docking energetics: if the same geometrically defined polar contacts are accessible at every receptor, a ligand capable of satisfying those requirements, whether steroidal or nonsteroidal, may produce comparable docking-derived energetic estimates across the panel. Selectivity in the pharmacological sense operates downstream of this initial pocket occupancy and is therefore invisible to the docking analysis by design.

#### 3.1.2. Steroidal vs. Nonsteroidal Binding Mode: Interaction Density, Diversity, and Thermodynamic Consequences

The endogenous reference ligands establish the baseline for what an optimized steroidal binding mode looks like. All five controls rely almost exclusively on conventional hydrogen bonds and alkyl/Pi-Alkyl contacts, with interaction-type diversity that is narrow but spatially precise. The sole exceptions are the Pi-Pi T-shaped contact of estradiol with Phe404 at ER and the extensive van der Waals network unique to the progesterone–PR complex, which encompasses nine residues (Val760, Met801, Met759, Phe778, Leu763, Gln725, Leu721, Trp755, and Gly722) as a discrete interaction category absent from the other four endogenous control profiles. Cortisol at GR presents the richest hydrogen-bonding network among all endogenous ligands, with six conventional hydrogen bonds (Gln570, Arg611, Leu563, Gln642, Thr739, and Asn564), which is consistent with its highest ΔG of all controls (−15.14 kcal/mol) and underscores the quantitative relationship between hydrogen-bond count and binding free energy within this receptor class.

YK11, despite sharing a steroidal tetracyclic framework with the endogenous controls, diverges from the endogenous binding template in a pharmacologically significant way. Its D-ring-fused butenolide moiety and C17 dioxolane group introduce additional polar functional groups absent in testosterone or progesterone, enabling hydrogen bonds with residues such as Gln642 and Thr739 at GR, and Val760 at PR, that are not contacted by the structurally simpler endogenous steroids. More importantly, the electron-rich aromatic character of the butenolide system generates Pi-Sigma interactions with Phe764 at AR and Pi-Sulfur contacts with Met807 at MR, interaction types that are entirely absent from the endogenous control profiles. This structural elaboration explains, at least in part, why YK11 achieves binding free energies at GR and PR that rival or exceed those of cortisol and progesterone: its scaffold satisfies the hydrophobic requirements of the binding pocket while introducing supplementary interaction types that provide stabilization beyond what the simpler endogenous scaffold achieves. The particularly favorable GR docking-derived binding energy of YK11 (ΔG = 0.35 kcal/mol relative to cortisol) is therefore not surprising but a predictable consequence of the corticosteroid-like physicochemical profile of the YK11 butenolide scaffold. This observation is relevant in the context of YK11’s broader pharmacological risk profile: rodent studies have associated YK11 with oxidative stress and mitochondrial dysfunction, and the possibility that its steroidal scaffold engages GR at a level comparable to the endogenous glucocorticoid may contribute to off-target endocrine effects that extend beyond androgen signaling.

Ostarine’s interaction profile is qualitatively distinct from both the steroidal controls and YK11. The most immediately apparent difference is the systematic presence of halogen (fluorine) bonds at every receptor—with Met745 at AR, Leu346/Glu353/Phe404 at ER, Leu721/Gly722 at PR, Leu563 at GR, and Phe941/Cys942 at MR—a class of directional non-covalent interaction entirely absent from the steroidal profiles. Although individual halogen bonds are energetically weaker than conventional hydrogen bonds, their consistent appearance across all five receptors indicates that the trifluoromethyl group contributes systematically to binding across the panel, partially compensating for the reduced hydrophobic contact density arising from ostarine’s smaller molecular volume relative to steroidal ligands. A second characteristic feature is the appearance of Pi-Pi aromatic stacking interactions—T-shaped contacts at ER (Phe404), GR (Phe623, Phe749), and MR (Phe829), and a Pi-Pi Stacked interaction at PR (Phe778)—arising from ostarine’s two conformationally unconstrained aromatic rings, which can adopt face-to-face or edge-to-face orientations that the fixed steroidal ring system cannot. The interaction-type diversity of ostarine reaches a maximum of seven distinct categories at PR, which is not evidence of stronger binding but of a more geometrically flexible ligand compensating through breadth rather than depth of interaction. This flexibility carries an entropic cost, reflected in ostarine’s consistently less favorable binding free energies relative to both YK11 and the endogenous controls across all five receptors.

A notable feature of the ostarine docking profile was the recurrent involvement of its trifluoromethyl group in halogen-bond-type contacts across all five receptor systems. These interactions were observed with different pocket-lining residues, including Met745 in AR, Leu346/Glu353/Phe404 in ER, Leu721/Gly722 in PR, Leu563 in GR, and Phe941/Cys942 in MR. These contacts’ recurrence suggests that the trifluoromethyl substituent may contribute to the ability of ostarine to maintain favorable receptor-pocket compatibility across structurally related steroid hormone receptor ligand-binding domains. From a medicinal chemistry perspective, this observation is relevant because the CF_3_ group combines strong lipophilicity, electron-withdrawing character, and directional interaction potential, allowing it to occupy hydrophobic subpockets while also forming polarizable contacts with suitable amino acid environments. In the context of SARM design, such interactions may be beneficial for AR binding, but may also contribute to broader receptor accommodation when conserved hydrophobic regions are present across related nuclear receptors. Therefore, the systematic CF_3_-associated contacts observed here support the idea that substituent-level interaction patterns, not only global docking scores, should be considered when optimizing nonsteroidal SARMs for receptor preference.

The torsional free energy term also provides useful context for the differences between the steroidal and nonsteroidal ligands. Across all five receptor systems, ostarine showed a higher torsional penalty (+1.79 kcal/mol) than YK11 (+1.19 kcal/mol) and the endogenous steroidal ligands, which ranged from +0.00 to +0.30 kcal/mol. This pattern is consistent with the greater conformational flexibility of the nonsteroidal arylpropionamide scaffold of ostarine compared with the more rigid tetracyclic steroidal frameworks of YK11 and the endogenous hormones. For example, at the androgen receptor, the difference in torsional penalty between ostarine and YK11 was 0.60 kcal/mol, while the total difference in predicted binding free energy between the two ligands was 2.49 kcal/mol. Thus, the torsional term accounts for approximately one quarter of the observed AutoDock-derived ΔG difference at AR, indicating that conformational cost contributes to, but does not fully explain, the weaker predicted binding of ostarine relative to YK11. The remaining difference is likely associated with intermolecular interaction energy and receptor-pocket complementarity.

#### 3.1.3. Destabilizing Contacts and Receptor-Specific Geometric Mismatch

Two instances of unfavorable contacts provide particularly instructive receptor-specific information. At MR, YK11 forms an acceptor-acceptor electrostatic clash between its C3 carbonyl oxygen and Phe829, the only destabilizing contact detected for YK11 across the entire receptor panel, which contributes to the thermodynamic penalty responsible for YK11’s comparatively less favorable MR docking-derived energy (ΔG = −12.28 kcal/mol) relative to GR (−14.79 kcal/mol) and PR (−14.03 kcal/mol). The position of Phe829 in the MR cavity is topologically distinct from the analogous positions in GR and PR, indicating that the suboptimal geometric fit of YK11’s A-ring carbonyl is a receptor-specific rather than scaffold-dependent limitation. At GR, ostarine generates a donor–donor repulsion between its hydroxyl group and Gln642. This is particularly instructive in direct comparison with YK11 at the same receptor, where Gln642 acts as a hydrogen-bond acceptor for the dioxolane oxygen of YK11, which is a stabilizing contact. The electronic reversal at this single residue (stabilizing for YK11, destabilizing for ostarine) illustrates how the same binding pocket position can contribute oppositely to the thermodynamics of two ligands as a function of local electronic geometry, and provides a structural explanation for the substantial difference in GR docking-derived energy between YK11 (−14.79 kcal/mol) and ostarine (−11.60 kcal/mol) that is not fully captured by global comparison of binding free energies alone.

### 3.2. Mechanistic Limitations of Flexible Docking in Predicting SARM Tissue Selectivity

The failure of the present docking analysis to reproduce the known tissue selectivity of ostarine reflects limitations that operate at levels beyond the scope of any docking protocol, including one employing flexible receptor residues as used here. The flexible docking setup in which eight active-site residues per receptor were defined as rotatable captures local side-chain mobility within the binding pocket and partially accounts for induced-fit effects at the immediate ligand-binding interface. This is a meaningful improvement over fully rigid models. However, it does not address the global conformational dynamics of the receptor that are most critical for SARM pharmacology, particularly the repositioning of helix-12 and the reorganization of the activation function-2 (AF-2) surface, which determine which coregulatory proteins are recruited upon ligand binding.

The mechanistic basis of SARM tissue selectivity cannot be inferred from ligand-binding pocket occupancy alone. Although receptor binding is required for activity, the functional outcome of AR modulation depends on ligand-induced receptor conformation and the subsequent recruitment or exclusion of transcriptional coregulators [62,63,64]. Experimental studies have shown that SARM-bound AR can assemble coregulator complexes that differ from those recruited by DHT-bound AR. In particular, SARM-dependent AR activation has been associated with altered p160/SRC-1 coactivator engagement, reduced recruitment of CBP/p300 and CARM1, and differential use of LXXLL motifs within p160 coactivators, supporting the concept that distinct AR ligands can produce different transcriptional outputs despite binding to the same receptor [64]. In parallel, AR activity may be negatively regulated by corepressors such as SMRT/NCOR2, which can interact with AR, inhibit AR N/C-terminal interaction, and compete with p160 coactivators [65].

Therefore, the role of coregulators in SARM pharmacology should be understood as a ligand- and cellular-context-dependent regulatory mechanism rather than as a simple consequence of receptor binding affinity. The present docking study does not quantify coregulator expression in muscle, prostate, or other androgen-responsive tissues and does not directly evaluate coactivator or corepressor recruitment. Instead, these experimentally established coregulatory mechanisms are cited to explain why ligand-binding-domain docking alone cannot predict tissue-selective functional responses. Docking can describe receptor-pocket compatibility, but it cannot determine whether a ligand-bound AR conformation will preferentially recruit coactivators, retain corepressors, alter chromatin engagement, or generate tissue-specific transcriptional programs [66].

Importantly, ligand-binding-domain docking captures only the initial recognition event between a small molecule and the receptor pocket. It does not determine whether the resulting ligand–receptor complex will behave as a full agonist, partial agonist, antagonist, or tissue-selective modulator. For SARMs, this distinction is particularly important because their pharmacological selectivity is not defined solely by preferential AR binding, but by the downstream transcriptional outcome generated after receptor occupancy. Two ligands may display comparable docking-derived energetic estimates for the same receptor while stabilizing different receptor conformations, exposing different AF-2 surfaces, altering N/C-terminal communication, and recruiting different combinations of coactivators and corepressors. Conversely, a ligand may show favorable docking compatibility with several steroid hormone receptor ligand-binding domains without necessarily producing functional activation of those receptors in cells or tissues. Therefore, the docking results presented in this study should be interpreted as evidence of receptor-pocket compatibility rather than as evidence of receptor activation, endocrine cross-reactivity, or tissue-level pharmacological selectivity.

The expectation that receptor conformational dynamics may influence SARM selectivity is supported by structural and functional studies of nuclear receptors and the androgen receptor [63,67]. In nuclear receptor ligand-binding domains, helix 12 is a central structural element of the activation function-2 (AF-2) surface. Upon agonist binding, helix 12 typically adopts a position that stabilizes the ligand-binding pocket and contributes to the formation of a hydrophobic coactivator-binding groove, whereas altered helix 12 positioning can impair coactivator recruitment or favor alternative coregulator interactions. In the androgen receptor, ligand binding has been shown to promote helix 12 repositioning and formation of an AF-2 surface capable of engaging coactivator motifs, while antagonist-bound conformations may alter this surface and support corepressor-related mechanisms. Structural studies have also demonstrated that ligands designed to interfere with helix 12 positioning can block ligand-dependent transactivation despite retaining receptor binding affinity. Therefore, receptor occupancy and binding-pocket compatibility alone are not sufficient to define the transcriptional outcome of ligand binding.

This distinction is particularly relevant for SARMs, whose functional selectivity is thought to depend not only on receptor binding, but also on ligand-specific receptor conformations, differential AF-2 surface presentation, N/C-terminal interactions, and tissue-dependent availability of coactivators and corepressors. Molecular dynamics studies of androgen receptor complexes further support the view that agonists and antagonists can stabilize different conformational ensembles, including differences in the mobility of ligand-binding domain regions that are not represented in a single docking pose [68].

A second limitation concerns the structural homology of the five receptors examined. The LBDs of AR, PR, and GR share extensive sequence identity and present ligand-binding cavities of similar hydrophobic character with geometrically conserved polar anchor residues, as described above. This structural relatedness is a well-established source of pharmacological cross-reactivity for steroidal and semi-steroidal ligands, and the docking results are entirely consistent with this expectation. Both YK11 and ostarine produce favorable ΔG values across the panel, not because they lack pharmacological selectivity but because their scaffolds are geometrically and chemically compatible with the shared structural features of these receptor pockets. The docking analysis identifies thermodynamic compatibility with a binding pocket, a necessary but far from sufficient condition for predicting the biological outcome of that occupancy.

An important methodological consideration is that the present analysis was performed exclusively using AutoDock 4.2 and its associated empirical free-energy scoring function. Although ab initio ligand optimization could provide more accurate isolated-ligand geometries and electronic descriptions, such calculations were not expected to substantially change the main interpretation of the present study, which depends primarily on comparative receptor-pocket accommodation within a single AutoDock 4.2 scoring and sampling framework rather than on high-level quantum-chemical ligand energetics. Therefore, the conclusions should not be interpreted as a universal assessment of all docking platforms or scoring functions. Alternative docking engines, such as AutoDock Vina, GOLD, Glide, or other commercial and open-source workflows, may differ in sampling algorithms, treatment of ligand flexibility, receptor flexibility, solvation terms, and scoring-function parametrization. Consequently, the numerical binding energies and even ligand ranking patterns may vary across methods. The main conclusion of the present study is therefore restricted to the AutoDock 4.2-based flexible docking protocol applied here: within this framework, receptor-binding energetics did not reproduce the functional tissue selectivity attributed to SARMs, particularly in the case of ostarine. Broader conclusions regarding docking as a general methodological class would require cross-platform benchmarking using multiple docking engines and, ideally, molecular dynamics and experimental validation.

The cluster analysis adds an additional methodological layer to the interpretation of the docking results. In all ligand-receptor systems, the selected lowest-energy pose belonged to cluster rank 1, indicating that the reported conformations were consistently derived from the top-ranked docking cluster. However, the population of these selected clusters differed markedly among ligands. The steroidal ligands, including YK11 and the endogenous reference hormones, generally showed higher selected cluster populations. This indicates better convergence toward the reported binding modes and supports the representativeness of these poses within the applied AutoDock 4.2 workflow. In contrast, ostarine consistently showed lower selected cluster populations across the receptor panel, suggesting a more dispersed docking behavior and reduced pose convergence. This pattern is consistent with the greater conformational flexibility and higher torsional penalty of the nonsteroidal ostarine scaffold. Therefore, although the ostarine poses were retained according to the lowest-energy criterion and belonged to cluster rank 1, their exact orientations should be interpreted more cautiously than those of the steroidal ligands.

A further limitation of the present study is the restricted size of the ligand dataset. Only two SARMs were included: ostarine, as a representative nonsteroidal SARM, and YK11, as a structurally distinct steroidal SARM-like ligand. Therefore, the findings should not be generalized to the entire chemically diverse SARM class. Ostarine was chosen because it is one of the best-characterized nonsteroidal SARMs, with substantial preclinical and clinical literature. YK11 was selected because it represents a more controversial steroidal scaffold with limited experimental validation, increasing visibility in nonmedical use, and potential concern regarding off-target steroid receptor interactions. Consequently, the present results should be interpreted as a case-based demonstration of discordance between AutoDock 4.2-derived receptor-binding energetics and functional SARM selectivity.

Beyond conventional docking, several computational approaches may provide a more complete assessment of SARM receptor selectivity. Molecular dynamics simulations can evaluate ligand-dependent receptor flexibility, helix 12 mobility, AF-2 surface remodeling, hydrogen-bond persistence, and conformational stability of receptor–ligand complexes over time. MM/PBSA and MM/GBSA approaches may further provide approximate post-docking estimates of binding free energy and residue-level energetic contributions, although their accuracy depends strongly on simulation quality, solvation treatment, and conformational sampling. More rigorous alchemical free-energy methods, such as free-energy perturbation and thermodynamic integration, can provide more quantitative estimates of relative ligand-binding free-energy estimates, but require carefully parameterized systems, extensive sampling, and substantial computational resources.

Other approaches may complement structure-based simulations. Pharmacophore modeling can identify ligand features associated with receptor recognition and may be useful for comparing scaffold classes within the SARM chemical space. Quantitative structure–activity relationship models and machine learning-based prediction frameworks can integrate molecular descriptors, receptor interaction fingerprints, experimental bioactivity data, and ADMET-related features to predict receptor preference or off-target risk. However, the reliability of such models depends on the availability of high-quality, receptor-specific experimental datasets, which remain limited for several SARMs, particularly for non-AR steroid hormone receptor interactions. Therefore, future computational studies of SARM selectivity would benefit from integrated workflows combining docking, molecular dynamics, free-energy calculations, pharmacophore modeling, and experimentally trained predictive models.

Future studies should expand the ligand panel to include additional SARMs from diverse chemical classes, such as arylpropionamides, quinolinones, hydantoins, bicyclic hydantoins, and other nonsteroidal AR modulators. Such an expanded dataset would allow a more robust assessment of whether the observed docking behavior is compound-specific, scaffold-dependent, or representative of a broader limitation in using docking-derived receptor affinity to infer functional SARM selectivity.

Taken together, the present findings support the following interpretation. For ostarine, the absence of a clearly preferential AR binding energy does not contradict its established pharmacological selectivity. It reveals that such selectivity does not originate at the step of initial LBD binding and pocket occupancy. The known tissue selectivity of ostarine, demonstrated in castrated rat models and in randomized clinical trials, is a post-binding, coregulator-mediated phenomenon that emerges from the specific AF-2 conformation ostarine stabilizes in different tissue environments, neither of which is captured by docking simulations, regardless of the degree of receptor flexibility modeled. The interaction analysis further demonstrates that ostarine achieves thermodynamic competence at each non-AR receptor by satisfying the same conserved polar anchor requirements as the endogenous hormones, using alternative chemical groups (cyano nitrogen, amide nitrogen, hydroxyl) and supplementary interaction types (halogen bonds, Pi-Pi stacking) that compensate for its smaller molecular volume. This is a structural explanation for why docking-derived binding free energies necessarily underestimate receptor specificity for nonsteroidal SARMs.

For YK11, the interpretive situation is more complex because of its steroidal scaffold and limited experimental characterization. In the present docking workflow, YK11 showed favorable predicted binding not only toward AR, but also toward GR and PR, suggesting that its scaffold is geometrically and physicochemically compatible with several steroid hormone receptor ligand-binding domains. However, these docking-derived affinities should not be interpreted as evidence of functional receptor activation, in vivo receptor occupancy, or a mechanistic basis for reported toxicological effects. Although experimental studies have reported mitochondrial dysfunction, oxidative stress, and neurochemical alterations after YK11 exposure, the present docking data do not establish that these effects are mediated through GR, PR, or any other non-AR steroid receptor pathway. Instead, the observed GR and PR compatibility should be regarded as a hypothesis-generating finding that warrants future experimental validation using receptor-binding assays, transactivation assays, coregulator recruitment studies, and cellular models relevant to YK11 toxicity.

### 3.3. Literature-Based Comparison with Available Experimental Binding Data

To place the docking-derived binding affinity estimates into an experimental context, we performed a literature-based search for direct in vitro binding data for ostarine/enobosarm/MK-2866/S-22 toward the five steroid hormone receptors investigated in this study. The results of this comparison are summarized in Table 6, and the corresponding supplementary literature-based comparison is provided in Supplementary Table S4. The available peer-reviewed literature provides experimental androgen receptor binding data for ostarine, with reported nanomolar AR affinity. In particular, ostarine/S-22 has been reported to bind the androgen receptor with a Ki of approximately 3.8 nM in competitive radioligand binding assays, whereas the AutoDock 4.2-derived Ki obtained in the present study for AR was 7.52 nM [69]. This represents approximate agreement within the same order of magnitude and provides a useful single-point reference for the docking protocol, although the two values should not be considered directly interchangeable because docking-derived Ki values are calculated from an empirical scoring function rather than measured under equilibrium binding conditions.

For the remaining receptor systems, namely ER, PR, GR, and MR, no peer-reviewed primary publications reporting direct experimental Ki, Kd, or IC50 values for ostarine were identified. Qualitative statements regarding receptor selectivity have been reported in the SARM literature, but these do not provide directly comparable quantitative binding constants for the non-AR steroid hormone receptors. Therefore, systematic numerical validation of the docking-derived Ki values across the full receptor panel could not be performed. This limitation does not invalidate the comparative docking analysis, but it requires that the non-AR docking results be interpreted as hypothesis-generating indicators of receptor-pocket compatibility rather than experimentally validated binding affinities.

The absence of publicly available quantitative cross-receptor binding data for ostarine also highlights a relevant gap in the pharmacological characterization of this compound. Future studies using competitive radioligand displacement assays, fluorescence polarization, surface plasmon resonance, isothermal titration calorimetry, or related orthogonal binding methods across a steroid hormone receptor panel would be necessary to determine whether the broad docking-compatible profile observed here translates into measurable non-AR receptor binding under experimental conditions. In addition, artificial intelligence and machine-learning-based approaches may further support this validation process by integrating docking outputs, structural descriptors, and experimental binding data to prioritize compounds with potential off-target steroid receptor interactions for subsequent experimental assessment [70].

## 4. Materials and Methods

### 4.1. Ligand Dataset Preparation

Two selective androgen receptor modulators (SARMs), YK11 and ostarine, were selected as the main ligands in the present study based on their relevance as performance-enhancing agents and their reported interaction with the androgen receptor. In addition, five endogenous steroid hormones—testosterone, estradiol, progesterone, cortisol, and aldosterone—were included as reference ligands for the corresponding nuclear receptors in order to enable comparative evaluation of binding affinity and receptor selectivity.

The three-dimensional (3D) structures of all ligands were retrieved from the PubChem database in SDF format. The endogenous hormones were used as reference compounds for androgen receptor (AR), estrogen receptor (ER), progesterone receptor (PR), glucocorticoid receptor (GR), and mineralocorticoid receptor (MR), respectively.

### 4.2. Preparation of Compounds for Molecular Docking

Ligands were prepared in their dominant neutral protonation states expected under physiological conditions. The endogenous steroid hormones, YK11, and ostarine were treated as neutral molecules during docking. No alternative tautomeric or ionization states were evaluated. This choice was made to maintain consistency across the ligand panel and because the investigated compounds do not contain strongly ionizable groups expected to carry a formal charge under physiological conditions.

Each ligand was imported into Avogadro 2.0.0, and its geometry was optimized using the “Optimize Geometry” function with the MMFF94 force field, which is commonly used for small organic molecules. The optimized structures were subsequently saved in MOL2 format. A higher-level quantum-mechanical geometry optimization was not performed because the purpose of this step was limited to obtaining chemically reasonable, strain-minimized starting conformations for subsequent AutoDock 4.2 docking. The study was designed as a comparative docking workflow rather than as a quantum-chemical evaluation of isolated ligand electronic structure.

The optimized structures were subsequently saved in MOL2 format. The resulting MOL2 files were then loaded into AutoDock Tools 1.5.7. Partial charges were assigned using the standard AutoDock Tools 1.5.7 workflow. Gasteiger charges were assigned to all ligand atoms after the addition of polar hydrogens and the detection of rotatable bonds. For receptor preparation, polar hydrogens were added, and Kollman united-atom charges were assigned according to the AutoDock 4.2 protocol. The prepared ligand and receptor structures were saved in PDBQT format, which was used as input for all docking simulations.

### 4.3. Target Receptor Preparation

The receptor structures were selected according to the following criteria: human origin, availability of an experimentally determined ligand-binding domain structure, presence of a co-crystallized endogenous ligand or agonist-bound conformation, acceptable crystallographic resolution for docking studies, and preservation of the canonical steroid hormone receptor ligand-binding pocket. Ligand-bound structures were prioritized because the present study aimed to compare the accommodation of YK11 and ostarine within biologically relevant steroid-binding cavities rather than to model ligand-induced receptor opening from an apo state. The selected structures, therefore, represent receptor conformations compatible with endogenous hormone binding and provide defined binding-site coordinates for targeted docking.

The selected structures included the human androgen receptor ligand-binding domain in complex with testosterone (PDB ID: 2AM9; 1.64 Å), human estrogen receptor alpha ligand-binding domain in complex with estradiol (PDB ID: 1A52; 2.80 Å), human progesterone receptor ligand-binding domain in complex with progesterone (PDB ID: 1A28; 1.80 Å), human glucocorticoid receptor ligand-binding domain in complex with cortisol (PDB ID: 4P6X; 2.50 Å), and human mineralocorticoid receptor ligand-binding domain in complex with aldosterone (PDB ID: 2AA2; 1.95 Å). These structures were chosen to provide a consistent panel of human steroid hormone receptor ligand-binding domains in agonist-compatible conformations. Although more recent structures are available for some receptor systems, they differ in ligand identity, mutations, stabilizing cofactors, antagonist-bound conformations, engineered constructs, or crystallographic conditions. Therefore, the selected structures were considered suitable for a standardized comparative docking workflow centered on endogenous hormone-binding pockets.

Initial preprocessing of the receptor structures was performed in UCSF ChimeraX, where non-essential molecules, including co-crystallized ligands and solvent molecules, were removed. The processed structures were then imported into AutoDock Tools 1.5.7. Polar hydrogen atoms were added, Kollman charges were assigned, and atom types were set according to the AutoDock 4 protocol. The prepared receptor structures were then saved in PDBQT format.

### 4.4. Flexible Docking Setup

A flexible docking protocol was applied in order to better account for local side-chain mobility within the ligand-binding domain of each receptor, as seen in Table 7 and Supplementary Table S1. For each receptor, selected amino acid residues from the ligand-binding pocket were defined as flexible based on their established involvement in ligand recognition and stabilization according to literature data. The number of flexible residues was limited to eight per receptor as a pragmatic compromise between incorporating local side-chain flexibility within the ligand-binding domain and maintaining computational tractability within the AutoDock 4.2 flexible receptor workflow. For each receptor, residues were selected based on their location within the crystallographic ligand-binding pocket, their proximity to the co-crystallized ligand or known steroid-binding region, and literature evidence supporting their involvement in ligand recognition, hydrogen bonding, hydrophobic stabilization, or steroid receptor selectivity. Priority was given to residues forming conserved polar anchoring interactions and residues lining the hydrophobic cavity that accommodates steroidal and nonsteroidal ligands. Thus, the flexible residue set was intended to capture the most relevant local side-chain adaptations at the ligand-binding interface rather than to model global receptor conformational dynamics. The remaining part of the receptor was kept rigid.

For the androgen receptor (PDB ID: 2AM9), the following residues were treated as flexible: Asn705, Leu701, Gln711, Trp741, Met745, Arg752, Phe764, and Met780. The grid box was centered at x = 25.461875, y = 4.32725, z = 3.38425.

For the estrogen receptor (PDB ID: 1A52), the flexible residues were Glu353, Leu387, Leu391, Trp393, Arg394, Phe404, Met421, and His524. The grid box was centered at x = 108.350625, y = 19.94175, z = 97.7305.

For the progesterone receptor (PDB ID: 1A28), the flexible residues included Leu718, Asn719, Leu721, Gln725, Trp755, Arg766, Phe778, and Thr894. The grid box center coordinates were x = 20.683125, y = 10.343125, z = 61.588875.

For the glucocorticoid receptor (PDB ID: 4P6X), the selected flexible residues were Asn564, Gln570, Arg611, Phe623, Ile629, Met639, Gln642, and Thr739. The grid box was centered at x = 6.7235, y = 29.859625, z = −6.80825.

For the mineralocorticoid receptor (PDB ID: 2AA2), the flexible residues were Ser767, Asn770, Gln776, Ser810, Arg817, Met852, Cys942, and Thr945. The grid box center coordinates were x = 18.2863, y = 72.3550, z = 19.5303.

For each receptor, the selected flexible residues were defined separately in AutoDock Tools, generating a flexible receptor file, while the remaining receptor structure was treated as rigid. This hybrid rigid–flexible setup allowed the simulation of limited induced-fit effects within the ligand-binding domain while maintaining computational efficiency. Alternative flexible residue sets were not systematically evaluated in the present study. Therefore, the possibility that absolute docking scores or ligand ranking patterns may be influenced by the selected flexible residue set cannot be excluded. However, the same standardized selection strategy was applied consistently across all receptor systems, allowing comparative interpretation within the defined AutoDock 4.2 workflow.

### 4.5. Molecular Docking Procedure

Docking simulations were performed using AutoDock 4.2 with the Lamarckian Genetic Algorithm (LGA). A targeted docking approach was used, with the grid box centered on the ligand-binding domain of each receptor based on the coordinates specified above. This setup ensured that docking was focused on the biologically relevant binding pocket rather than the entire receptor surface.

For each receptor, the grid box was centered on the crystallographic ligand-binding domain, as defined by the position of the co-crystallized ligand or the known steroid-binding pocket. Grid maps were generated using dimensions of 60 × 60 × 60 grid points with a spacing of 0.375 Å in all three directions. The grid dimensions were selected to fully cover the ligand-binding cavity and the selected flexible residues while avoiding unnecessary expansion into solvent-exposed regions.

The docking parameters included 100 runs per ligand–receptor pair, a maximum of 25,000,000 energy evaluations, a population size of 150, a mutation rate of 0.02, and a crossover rate of 0.8. The best binding pose for each ligand–receptor complex was selected based on the lowest binding free energy (ΔG, kcal/mol). Docked conformations were clustered using a root-mean-square deviation (RMSD) tolerance of 2.0 Å. For each ligand–receptor pair, the reported binding energy and docking-derived inhibition constant correspond to the best-ranked lowest-energy pose obtained from 100 independent docking runs. These values were used for comparative ranking within the applied AutoDock 4.2 workflow and should not be interpreted as statistical mean binding energies.

Because the study was designed as a comparative AutoDock 4.2-based analysis, no alternative docking engines or scoring functions were applied. Therefore, the reported binding energies and inhibition constants should be interpreted within the assumptions and limitations of the AutoDock 4.2 scoring function.

### 4.6. Docking Analysis and Visualization

The obtained docking results were evaluated using the calculated binding free energy (ΔG), inhibition constant (Ki), intermolecular energy, total internal energy, torsional free energy, unbound energy, and the number of generated conformations. The comparative interpretation of these parameters was used to assess the receptor-binding profiles of YK11 and ostarine across the five nuclear receptors.

Ligand–receptor interactions were analyzed in UCSF ChimeraX 1.12, focusing on the spatial localization of ligands within the ligand-binding domain and their accommodation within the receptor cavity. Particular attention was given to the general binding behavior of the ligands in relation to the selected flexible residues and the overall receptor pocket environment.

### 4.7. Validation of the Docking Protocol

To ensure the reliability of the docking procedure, the protocol was validated by docking the endogenous ligands (testosterone, estradiol, progesterone, cortisol, and aldosterone) into their respective receptor structures. The obtained binding poses were localized within the ligand-binding domain and exhibited energetically favorable interactions consistent with the known binding sites of these receptors. This confirms that the applied docking setup is suitable for comparative analysis of ligand-receptor interactions across the studied nuclear receptor systems.

To further validate the docking protocol, re-docking of each co-crystallized endogenous ligand was performed into its corresponding receptor binding site using the same grid box coordinates, flexible residue settings, and docking parameters as those applied in the main docking experiments. The results are shown in Table 8.

The re-docking results demonstrated that the applied docking protocol was able to reproduce the experimentally observed binding orientations of the co-crystallized endogenous ligands within the corresponding receptor ligand-binding domains. The calculated RMSD values ranged from 1.05 Å to 1.65 Å, remaining below the commonly accepted threshold of 2.0 Å for successful docking pose reproduction. The lowest RMSD value was observed for progesterone in the progesterone receptor complex (1.05 Å), while the highest value was obtained for estradiol in the estrogen receptor complex (1.65 Å). These results indicate that the selected grid box coordinates, flexible residue settings, and docking parameters were suitable for recovering the crystallographic ligand-binding modes across the investigated steroid hormone receptor panel. Therefore, the docking protocol was considered appropriate for the subsequent comparative analysis of YK11 and ostarine binding profiles.

RMSD values below 2.0 Å were considered indicative of acceptable reproduction of the crystallographic binding mode, whereas values above this threshold were interpreted as reflecting reduced pose reproducibility within the applied AutoDock 4.2 flexible docking workflow. This validation step was used to assess the ability of the protocol to recover the experimentally observed ligand orientation within each steroid hormone receptor ligand-binding domain.

## 5. Conclusions

The methodological conclusion of this analysis is that, within the AutoDock 4.2-based flexible docking protocol applied in this study, docking-derived binding energies were insufficient as standalone predictors of SARM tissue selectivity. The interaction profiles and binding free energies generated by this workflow provide useful descriptions of the thermodynamic compatibility between ligand scaffolds and receptor ligand-binding pockets, and they offer structural rationalizations for binding affinity differences within and across the receptor panel. However, they should not be interpreted as direct indicators of functional selectivity. Importantly, because only one docking engine and scoring function were used, the present findings should be regarded as method-specific rather than as a universal limitation of all docking approaches. The present analysis, therefore, demonstrates a discordance between AutoDock 4.2-derived receptor-binding energetics and the functional tissue-selective pharmacology attributed to SARMs, rather than establishing a general limitation of all docking methodologies. Because the present ligand dataset was limited to one steroidal SARM-like compound and one nonsteroidal SARM, the conclusions should be interpreted as compound-specific and hypothesis-generating rather than as broadly generalizable to the entire SARM chemical class.

Future studies addressing SARM selectivity computationally should include cross-platform docking comparisons using alternative docking engines and scoring functions, followed by molecular dynamics simulations to assess receptor-specific conformational responses to ligand binding. In addition, coactivator recruitment modeling using AF-2 surface ensembles, in vitro transactivation assays in cell lines with defined coregulator expression profiles, reporter gene assays in muscle versus prostate cells, and in vivo receptor occupancy studies remain essential approaches for characterizing SARM selectivity. The present analysis maps the thermodynamic landscape of SARM-nuclear receptor interactions across a biologically relevant receptor panel and, in doing so, identifies both the structural basis for the observed cross-receptor binding affinity and the mechanistic reasons why that affinity does not translate directly into predictions of biological selectivity.

## Figures and Tables

**Table 1:** Molecular docking results of YK11, ostarine, and testosterone with the androgen receptor (PDB ID: 2AM9).

Ligand ID	YK11	Ostarine	Testosterone
Selected cluster rank	1	1	1
Selected cluster population	52	2	88
Binding energy, kcal/mol	−13.57	−11.08	−13.42
Inhibition Constant, Ki	113.14 pM	7.52 nM	145.77 pM
Intermolecular Energy, kcal/mol	−14.76	−12.87	−13.42
Total Internal energy, kcal/mol	+3.18 × 10^5^	+2.45 × 10^5^	+2.45 × 10^5^
Torsional Free Energy, kcal/mol	+1.19	+1.79	+0.00
Unbound Energy, kcal/mol	+3.18 × 10^5^	+2.45 × 10^5^	+2.45 × 10^5^

**Table 2:** Molecular docking results of YK11, ostarine, and estradiol with the estrogen receptor (PDB ID: 1A52).

Ligand ID	YK11	Ostarine	Estradiol
Selected cluster rank	1	1	1
Selected cluster population	7	4	59
Binding energy, kcal/mol	−11.32	−10.42	−10.88
Inhibition Constant, Ki	5.02 nM	22.99 nM	10.67 nM
Intermolecular Energy, kcal/mol	−12.52	−12.21	−10.88
Total Internal energy, kcal/mol	−6.56	−11.55	−12.10
Torsional Free Energy, kcal/mol	+1.19	+1.79	+0.00
Unbound Energy, kcal/mol	−6.56	−11.55	−12.10

**Table 3:** Molecular docking results of YK11, ostarine, and progesterone with the progesterone receptor (PDB ID: 1A28).

Ligand ID	YK11	Ostarine	Progesterone
Selected cluster rank	1	1	1
Selected cluster population	47	8	52
Binding energy, kcal/mol	−14.03	−12.05	−13.04
Inhibition Constant, Ki	51.93 pM	1.46 nM	277.38 pM
Intermolecular Energy, kcal/mol	−15.22	−13.84	−13.34
Total Internal energy, kcal/mol	−15.65	−15.30	−14.68
Torsional Free Energy, kcal/mol	+1.19	+1.79	+0.30
Unbound Energy, kcal/mol	−15.65	−15.30	−14.68

**Table 4:** Molecular docking results of YK11, ostarine, and cortisol with the glucocorticoid receptor (PDB ID: 4P6X).

Ligand ID	YK11	Ostarine	Cortisol
Selected cluster rank	1	1	1
Selected cluster population	47	1	39
Binding energy, kcal/mol	−14.79	−11.60	−15.14
Inhibition Constant, Ki	14.32 pM	3.12 nM	7.96 pM
Intermolecular Energy, kcal/mol	−15.99	−13.39	−15.44
Total Internal energy, kcal/mol	−12.37	−14.34	−12.17
Torsional Free Energy, kcal/mol	+1.19	+1.79	+0.30
Unbound Energy, kcal/mol	−12.37	−14.34	−12.17

**Table 5:** Molecular docking results of YK11, ostarine, and aldosterone with the mineralcorticoid receptor (PDB ID: 2AA2).

Ligand ID	YK11	Ostarine	Aldosterone
Selected cluster rank	1	1	1
Selected cluster population	69	9	66
Binding energy, kcal/mol	−12.28	−11.57	−13.55
Inhibition Constant, Ki	989.11 pM	3.31 nM	117.20 pM
Intermolecular Energy, kcal/mol	−13.48	−13.36	−13.85
Total Internal energy, kcal/mol	−9.36	−7.93	−9.66
Torsional Free Energy, kcal/mol	+1.19	+1.79	+0.30
Unbound Energy, kcal/mol	−9.36	−7.93	−9.66

**Table 6:** Literature-based comparison between AutoDock 4.2-derived inhibition constants and available experimental in vitro binding data for ostarine/enobosarm/MK-2866/S-22 across the steroid hormone receptor panel.

Ligand	Receptor	AutoDock 4.2-Derived Ki in the Present Study	Available Experimental Binding Data	Assay Type/ Receptor System	Source	Interpretation
Ostarine/enobosarm/MK-2866/S-22	AR/NR3C4	7.52 nM	Ki ≈ 3.8 nM	Competitive radioligand binding; androgen receptor binding assays reported in the SARM medicinal chemistry literature	[67]	Experimental and docking-derived values are within the same nanomolar range. This provides partial, single-point support for the order-of-magnitude accuracy of the AutoDock 4.2-derived AR estimate, but not full validation of the whole receptor panel.
Ostarine/enobosarm/MK-2866/S-22	ER/ESR1/ESR2	22.99 nM	No peer-reviewed direct Ki, Kd, or IC50 binding value identified	Not available	No primary peer-reviewed direct binding source identified in the searched literature	No numerical experimental comparison is possible. The docking result should be interpreted as a computational hypothesis rather than a validated ER binding estimate.
Ostarine/enobosarm/MK-2866/S-22	PR/PGR/NR3C3	1.46 nM	No peer-reviewed direct Ki, Kd, or IC50 binding value identified	Not available	No primary peer-reviewed direct binding source identified in the searched literature	No numerical experimental comparison is possible. The docking result should be interpreted as a computational hypothesis rather than a validated PR binding estimate.
Ostarine/enobosarm/MK-2866/S-22	GR/NR3C1	3.12 nM	No peer-reviewed direct Ki, Kd, or IC50 binding value identified	Not available	No primary peer-reviewed direct binding source identified in the searched literature	No numerical experimental comparison is possible. The docking result should be interpreted as a computational hypothesis rather than a validated GR binding estimate.
Ostarine/enobosarm/MK-2866/S-22	MR/NR3C2	3.31 nM	No peer-reviewed direct Ki, Kd, or IC50 binding value identified	Not available	No primary peer-reviewed direct binding source identified in the searched literature	No numerical experimental comparison is possible. The docking result should be interpreted as a computational hypothesis rather than a validated MR binding estimate.

**Table 7:** Flexible receptor residues selected for the molecular docking protocol across steroid hormone nuclear receptors.

Receptor	PDB ID	Flexible Residues	Rationale
AR	2AM9	Asn705, Leu701, Gln711, Trp741, Met745, Arg752, Phe764, Met780	Key ligand-binding pocket residues involved in polar and hydrophobic stabilization
ER	1A52	Glu353, Leu387, Leu391, Trp393, Arg394, Phe404, Met421, His524	Pocket-lining residues relevant for ligand accommodation
PR	1A28	Leu718, Asn719, Leu721, Gln725, Trp755, Arg766, Phe778, Thr894	Residues contributing to steroid ligand recognition
GR	4P6X	Asn564, Gln570, Arg611, Phe623, Ile629, Met639, Gln642, Thr739	Polar and hydrophobic residues shaping the binding cavity
MR	2AA2	Ser767, Asn770, Gln776, Ser810, Arg817, Met852, Cys942, Thr945	Conserved steroid receptor pocket residues

**Table 8:** Re-docking validation of the docking protocol.

Receptor	PDB ID	Co-Crystallized Ligand	Re-Docking Binding Energy	RMSD
AR	2AM9	Testosterone	−13.42 kcal/mol	1.10 Å
ER	1A52	Estradiol	−10.88 kcal/mol	1.65 Å
PR	1A28	Progesterone	−13.04 kcal/mol	1.05 Å
GR	4P6X	Cortisol	−15.14 kcal/mol	1.35 Å
MR	2AA2	Aldosterone	−13.55 kcal/mol	1.20 Å

## Data Availability

The original contributions presented in this study are included in the article and Supplementary Materials. Further inquiries can be directed to the corresponding authors.

## Supplementary Materials

The following supporting information can be downloaded at: https://www.mdpi.com/article/10.3390/ijms27135765/s1.

## References

[1] Van-Duyne G., Blair I.A., Sprenger C., Moiseenkova-Bell V., Plymate S., Penning T.M. (2023). The androgen receptor. Vitam. Horm..

[2] Naamneh Elzenaty R., du Toit T., Flück C.E. (2022). Basics of androgen synthesis and action. Best Pract. Res. Clin. Endocrinol. Metab..

[3] Vasiliou S.K., Diamandis E.P. (2019). Androgen receptor: A promising therapeutic target in breast cancer. Crit. Rev. Clin. Lab Sci..

[4] Dalal K., Roshan-Moniri M., Sharma A., Li H., Ban F., Hessein M., Hsing M., Singh K., LeBlanc E., Dehm S. (2014). Selectively targeting the DNA-binding domain of the androgen receptor as a prospective therapy for prostate cancer. J. Biol. Chem..

[5] Gerald T., Raj G. (2022). Testosterone and the Androgen Receptor. Urol. Clin. N. Am..

[6] Khan A.F., Karami S., Peidl A.S., Waiters K.D., Babajide M.F., Bawa-Khalfe T. (2023). Androgen Receptor in Hormone Receptor-Positive Breast Cancer. Int. J. Mol. Sci..

[7] Asangani I., Blair I.A., Van Duyne G., Hilser V.J., Moiseenkova-Bell V., Plymate S., Sprenger C., Wand A.J., Penning T.M. (2021). Using biochemistry and biophysics to extinguish androgen receptor signaling in prostate cancer. J. Biol. Chem..

[8] Rifaldi F., Tortorella A., Sporeni S., Lanzetta I., Figini S., Naspro R.L.J., Lancia A., Montagna B., Secondino S., Pedrazzoli P. (2025). Novel androgen receptor inhibitors in prostate cancer: What do we know so far?. Crit. Rev. Oncol. Hematol..

[9] Lee D.H., Kong S.H., Jang H.N., Ahn C.H., Lim S.G., Lee Y.A., Kim S.W., Kim J.H. (2022). Association of androgen excess and bone mineral density in women with classical congenital adrenal hyperplasia with 21-hydroxylase deficiency. Arch. Osteoporos..

[10] Santi D., Spaggiari G., Gilioli L., Potì F., Simoni M., Casarini L. (2018). Molecular basis of androgen action on human sexual desire. Mol. Cell. Endocrinol..

[11] Liu J., Chin-Yee B., Ho J., Lazo-Langner A., Chin-Yee I.H., Iansavitchene A., Hsia C.C. (2025). Diagnosis, management, and outcomes of drug-induced erythrocytosis: A systematic review. Blood Adv..

[12] Solomon Z.J., Mirabal J.R., Mazur D.J., Kohn T.P., Lipshultz L.I., Pastuszak A.W. (2019). Selective Androgen Receptor Modulators: Current Knowledge and Clinical Applications. Sex. Med. Rev..

[13] Bond P., Smit D.L., Verdegaal T., de Ronde W. (2025). Selective androgen receptor modulators: A critical appraisal. Front. Endocrinol..

[14] Kang J., Chen R., Tharakan T., Minhas S. (2022). Novel androgen therapies including selective androgen receptor modulators. Best Pract. Res. Clin. Endocrinol. Metab..

[15] Bhasin S., Krishnan V., Storer T.W., Steiner M., Dobs A.S. (2023). Androgens and Selective Androgen Receptor Modulators to Treat Functional Limitations Associated With Aging and Chronic Disease. J. Gerontol. A Biol. Sci. Med. Sci..

[16] Narayanan R., Coss C.C., Dalton J.T. (2018). Development of selective androgen receptor modulators (SARMs). Mol. Cell. Endocrinol..

[17] Fonseca G.W.P.D., Dworatzek E., Ebner N., Von Haehling S. (2020). Selective androgen receptor modulators (SARMs) as pharmacological treatment for muscle wasting in ongoing clinical trials. Expert Opin. Investig. Drugs.

[18] Vasireddi N., Hahamyan H.A., Gould H.P., Gregory A.J.M., Gausden E.B., Dodson C.C., Voos J.E., Calcei J.G. (2025). Athlete Selective Androgen Receptor Modulators Abuse: A Systematic Review. Am. J. Sports Med..

[19] Wen J., Syed B., Leapart J., Shehabat M., Ansari U., Akhtar M., Razick D., Pai D. (2025). Selective Androgen Receptor Modulators (SARMs) Effects on Physical Performance: A Systematic Review of Randomized Control Trials. Clin. Endocrinol..

[20] Borecki R., Byczkiewicz P., Słowikowska-Hilczer J. (2025). Selective androgen receptor modulators (SARMs)—Potential anabolic drugs for the treatment of cachexia and frailty syndrome. Endokrynol. Pol..

[21] Leciejewska N., Pruszyńska-Oszmałek E., Nogowski L., Sassek M., Strowski M.Z., Kołodziejski P.A. (2023). Sex-specific cytotoxicity of ostarine in cardiomyocytes. Mol. Cell. Endocrinol..

[22] Vasilev V., Boyadjiev N., Hrischev P., Gerginska F., Delchev S., Arabadzhiyska D., Komrakova M., Boeker K.O., Schilling A.F., Georgieva K. (2024). Ostarine blunts the effect of endurance training on submaximal endurance in rats. Naunyn Schmiedebergs Arch. Pharmacol..

[23] Nishikawa H., Goto M., Fukunishi S., Asai A., Nishiguchi S., Higuchi K. (2021). Cancer Cachexia: Its Mechanism and Clinical Significance. Int. J. Mol. Sci..

[24] Zilbermint M.F., Dobs A.S. (2009). Nonsteroidal selective androgen receptor modulator Ostarine in cancer cachexia. Future Oncol..

[25] Leciejewska N., Kołodziejski P.A., Sassek M., Nogowski L., Małek E., Pruszyńska-Oszmałek E. (2022). Ostarine-Induced Myogenic Differentiation in C2C12, L6, and Rat Muscles. Int. J. Mol. Sci..

[26] Taoussi O., Bambagiotti G., Gameli P.S., Daziani G., Tavoletta F., Tini A., Basile G., Lo Faro A.F., Carlier J. (2024). In Vitro and In Vivo Human Metabolism of Ostarine, a Selective Androgen Receptor Modulator and Doping Agent. Int. J. Mol. Sci..

[27] Komrakova M., Nagel J., Hoffmann D.B., Lehmann W., Schilling A.F., Sehmisch S. (2020). Effect of Selective Androgen Receptor Modulator Enobosarm on Bone Healing in a Rat Model for Aged Male Osteoporosis. Calcif. Tissue Int..

[28] Bedi H., Hammond C., Sanders D., Yang H.M., Yoshida E.M. (2021). Drug-Induced Liver Injury from Enobosarm (Ostarine), a Selective Androgen Receptor Modulator. ACG Case Rep. J..

[29] Walpurgis K., Rubio A., Wagener F., Krug O., Knoop A., Görgens C., Guddat S., Thevis M. (2020). Elimination profiles of microdosed ostarine mimicking contaminated products ingestion. Drug Test. Anal..

[30] Jendrzejewska I., Cehlarik L., Goryczka T., Pietrasik E., Pawlik N., Jampilek J. (2025). Rapid detection of illegal selective androgen receptor modulators in unregistered supplements using a combination of selected solid-state analytical methods. ADMET DMPK.

[31] Van Wagoner R.M., Eichner A., Bhasin S., Deuster P.A., Eichner D. (2017). Chemical Composition and Labeling of Substances Marketed as Selective Androgen Receptor Modulators and Sold via the Internet. JAMA.

[32] Hansson A., Knych H., Stanley S., Thevis M., Bondesson U., Hedeland M. (2015). Characterization of equine urinary metabolites of selective androgen receptor modulators (SARMs) S1, S4 and S22 for doping control purposes. Drug Test. Anal..

[33] Thevis M., Schänzer W. (2018). Detection of SARMs in doping control analysis. Mol. Cell. Endocrinol..

[34] Lee S.J., Gharbi A., Shin J.E., Jung I.D., Park Y.M. (2021). Myostatin inhibitor YK11 as a preventative health supplement for bacterial sepsis. Biochem. Biophys. Res. Commun..

[35] Wang R., Zhong Y., Du Q., Zhao C., Wang Y., Pan J. (2025). YK11 promotes osteogenic differentiation of BMSCs and repair of bone defects. J. Mol. Endocrinol..

[36] Yatsu T., Kusakabe T., Kato K., Inouye Y., Nemoto K., Kanno Y. (2018). Selective Androgen Receptor Modulator, YK11, Up-Regulates Osteoblastic Proliferation and Differentiation in MC3T3-E1 Cells. Biol. Pharm. Bull..

[37] Dahleh M.M.M., Bortolotto V.C., Boeira S.P., Segat H.J., Guerra G.P., Prigol M. (2024). From gains to gaps? How Selective Androgen Receptor Modulator (SARM) YK11 impact hippocampal function: In silico, in vivo, and ex vivo perspectives. Chem. Biol. Interact..

[38] Dahleh M.M.M., Bortolotto V.C., Guerra G.P., Boeira S.P., Prigol M. (2023). YK11 induces oxidative stress and mitochondrial dysfunction in hippocampus: The interplay between a selective androgen receptor modulator (SARM) and exercise. J. Steroid Biochem. Mol. Biol..

[39] Wiacek M., Zubrzycki I.Z. (2026). Anabolic-Androgenic Steroids Revisited: Structural Biology, Receptor Signaling, and Mechanisms of Anabolic-Androgenic Dissociation. Int. J. Mol. Sci..

[40] Bohlin K.P., Pohanka A., Andersson A., Villén T., Ekström L. (2024). Detection of anabolic agents including selective androgen receptor modulators in samples outside of sport. Drug Test. Anal..

[41] Machek S.B., Cardaci T.D., Wilburn D.T., Willoughby D.S. (2020). Considerations, possible contraindications, and potential mechanisms for deleterious effect in recreational and athletic use of selective androgen receptor modulators (SARMs) in lieu of anabolic androgenic steroids: A narrative review. Steroids.

[42] Vasireddi N., Hahamyan H., Salata M.J., Karns M., Calcei J.G., Voos J.E., Apostolakos J.M. (2025). Emerging Use of BPC-157 in Orthopaedic Sports Medicine: A Systematic Review. HSS J..

[43] Vignali J.D., Pak K.C., Beverley H.R., DeLuca J.P., Downs J.W., Kress A.T., Sadowski B.W., Selig D.J. (2023). Systematic Review of Safety of Selective Androgen Receptor Modulators in Healthy Adults: Implications for Recreational Users. J. Xenobiot..

[44] Khan S., Fackler J., Gilani A., Murphy S., Polintan L. (2022). Selective Androgen Receptor Modulator Induced Hepatotoxicity. Cureus.

[45] Leciejewska N., Jędrejko K., Gómez-Renaud V.M., Manríquez-Núñez J., Muszyńska B., Pokrywka A. (2024). Selective androgen receptor modulator use and related adverse events including drug-induced liver injury: Analysis of suspected cases. Eur. J. Clin. Pharmacol..

[46] Kintz P. (2022). The forensic response after an adverse analytical finding (doping) involving a selective androgen receptor modulator (SARM) in human athlete. J. Pharm. Biomed. Anal..

[47] Demangone M.R., Abi Karam K.R., Li J. (2024). Selective Androgen Receptor Modulators Leading to Liver Injury: A Case Report. Cureus.

[48] Mertens J.E., Bömmer M.T.C., Regier M.B., Gabriëls G., Pavenstädt H., Grünewald I., Horvath J., Trebicka J., Schmidt H., Schlevogt B. (2024). Liver Injury after Selective Androgen Receptor Modulator Intake: A Case Report and Review of the Literature. Z. Gastroenterol..

[49] Lee B.K., Park B.B., Bower R.J. (2023). Selective Androgen Receptor Modulator-Induced Liver Injury in Active Duty Male. Mil. Med..

[50] Mohideen H., Hussain H., Dahiya D.S., Wehbe H. (2023). Selective Androgen Receptor Modulators: An Emerging Liver Toxin. J. Clin. Transl. Hepatol..

[51] Weinblatt D., Roy S. (2022). Drug-Induced Liver Injury Secondary to Enobosarm: A Selective Androgen Receptor Modulator. J. Med. Cases.

[52] Christiansen A.R., Lipshultz L.I., Hotaling J.M., Pastuszak A.W. (2020). Selective androgen receptor modulators: The future of androgen therapy?. Transl. Androl. Urol..

[53] Kanno Y., Ota R., Someya K., Kusakabe T., Kato K., Inouye Y. (2013). Selective androgen receptor modulator, YK11, regulates myogenic differentiation of C2C12 myoblasts by follistatin expression. Biol. Pharm. Bull..

[54] Kanno Y., Hikosaka R., Zhang S.Y., Inoue Y., Nakahama T., Kato K., Yamaguchi A., Tominaga N., Kohra S., Arizono K. (2011). (17α,20E)-17,20-[(1-methoxyethylidene)bis(oxy)]-3-oxo-19-norpregna-4,20-diene-21-carboxylic acid methyl ester (YK11) is a partial agonist of the androgen receptor. Biol. Pharm. Bull..

[55] Piper T., Dib J., Putz M., Fusshöller G., Pop V., Lagojda A., Kuehne D., Geyer H., Schänzer W., Thevis M. (2018). Studies on the in vivo metabolism of the SARM YK11: Identification and characterization of metabolites potentially useful for doping controls. Drug Test. Anal..

[56] Kim J., Wu D., Hwang D.J., Miller D.D., Dalton J.T. (2005). The para substituent of S-3-(phenoxy)-2-hydroxy-2-methyl-N-(4-nitro-3-trifluoromethyl-phenyl)-propionamides is a major structural determinant of in vivo disposition and activity of selective androgen receptor modulators. J. Pharmacol. Exp. Ther..

[57] Gao W., Reiser P.J., Coss C.C., Phelps M.A., Kearbey J.D., Miller D.D., Dalton J.T. (2005). Selective androgen receptor modulator treatment improves muscle strength and body composition and prevents bone loss in orchidectomized rats. Endocrinology.

[58] Kearbey J.D., Gao W., Narayanan R., Fisher S.J., Wu D., Miller D.D., Dalton J.T. (2007). Selective Androgen Receptor Modulator (SARM) treatment prevents bone loss and reduces body fat in ovariectomized rats. Pharm. Res..

[59] Crawford J., Prado C.M., Johnston M.A., Gralla R.J., Taylor R.P., Hancock M.L., Dalton J.T. (2016). Study Design and Rationale for the Phase 3 Clinical Development Program of Enobosarm, a Selective Androgen Receptor Modulator, for the Prevention and Treatment of Muscle Wasting in Cancer Patients (POWER Trials). Curr. Oncol. Rep..

[60] Zazeri G., Povinelli A.P.R., Le Duff C.S., Tang B., Cornelio M.L., Jones A.M. (2020). Synthesis and Spectroscopic Analysis of Piperine- and Piperlongumine-Inspired Natural Product Scaffolds and Their Molecular Docking with IL-1β and NF-κB Proteins. Molecules.

[61] Zazeri G., Povinelli A.P.R., Pavan N.M., Jones A.M., Ximenes V.F. (2023). Solvent-Induced Lag Phase during the Formation of Lysozyme Amyloid Fibrils Triggered by Sodium Dodecyl Sulfate: Biophysical Experimental and In Silico Study of Solvent Effects. Molecules.

[62] Agoulnik I.U., Weigel N.L. (2008). Androgen receptor coactivators and prostate cancer. Adv. Exp. Med. Biol..

[63] Hodgson M.C., Shen H.C., Hollenberg A.N., Balk S.P. (2008). Structural basis for nuclear receptor corepressor recruitment by antagonist-liganded androgen receptor. Mol. Cancer Ther..

[64] Baek S.H., Ohgi K.A., Nelson C.A., Welsbie D., Chen C., Sawyers C.L., Rose D.W., Rosenfeld M.G. (2006). Ligand-specific allosteric regulation of coactivator functions of androgen receptor in prostate cancer cells. Proc. Natl. Acad. Sci. USA.

[65] Rahman M., Miyamoto H., Chang C. (2004). Androgen Receptor Coregulators in Prostate Cancer: Mechanisms and Clinical Implications. Clin. Cancer Res..

[66] Liao G., Chen L.Y., Zhang A., Godavarthy A., Xia F., Ghosh J.C., Li H., Chen J.D. (2003). Regulation of androgen receptor activity by the nuclear receptor corepressor SMRT. J. Biol. Chem..

[67] Kallenberger B.C., Love J.D., Chatterjee V.K., Schwabe J.W. (2003). A dynamic mechanism of nuclear receptor activation and its perturbation in a human disease. Nat. Struct. Biol..

[68] Gim H.J., Park J., Jung M.E., Houk K.N. (2021). Conformational dynamics of androgen receptors bound to agonists and antagonists. Sci. Rep..

[69] Mohler M.L., Bohl C.E., Jones A., Coss C.C., Narayanan R., He Y., Hwang D.J., Dalton J.T., Miller D.D. (2009). Nonsteroidal selective androgen receptor modulators (SARMs): Dissociating the anabolic and androgenic activities of the androgen receptor for therapeutic benefit. J. Med. Chem..

[70] Mihaylova S.G., Lambev M.K., Bekyarov P.S., Georgieva D.D., Dangova M.G., Tsvetkova A.Z. (2026). Application of artificial intelligence in searching for new approaches to fight antimicrobial resistance. Farmacia.

