# Supplementary material for: Limitations of Molecular Docking in Predicting the Selectivity of Selective Androgen Receptor Modulators (SARMs): A Comparative Study of YK11 and Ostarine Across Five Nuclear Receptors
Source: Int J Mol Sci. 2026 Jun 26;27(13):5765. doi: 10.3390/ijms27135765 (PMC13361875; doi:10.3390/ijms27135765)
Supplement: Supplementary file 1 [file ijms-27-05765-s001.zip › S1_table.pdf]

Table S1. Grid box coordinates, flexible residue sets, and common AutoDock 4.2 docking parameters used for each receptor system.

Receptor	PDB ID	Flexible residues	Selection rationale	Grid center X	Grid center Y	Grid center Z	Grid dimensions	Grid spacing	Common docking parameters
AR	2AM9	Asn705, Leu701, Gln711, Trp741, Met745, Arg752, Phe764, Met780	Selected ligand-binding pocket residues involved in polar anchoring and hydrophobic stabilization of androgen/SARM ligands.	25.461875	4.32725	3.38425	60 x 60 x 60	0.375	AutoDock 4.2; Lamarckian Genetic Algorithm; 100 docking runs; 25,000,000 energy evaluations; population size 150; mutation rate 0.02; crossover rate 0.8; RMSD clustering tolerance 2.0 Å
ER	1A52	Glu353, Leu387, Leu391, Trp393, Arg394, Phe404, Met421, His524	Selected pocket-lining residues involved in estrogen recognition, polar anchoring, aromatic contacts, and hydrophobic ligand accommodation.	108.350625	19.94175	97.7305	60 x 60 x 60	0.375	AutoDock 4.2; Lamarckian Genetic Algorithm; 100 docking runs; 25,000,000 energy evaluations; population size 150; mutation rate 0.02; crossover rate 0.8; RMSD clustering tolerance 2.0 Å
PR	1A28	Leu718, Asp719, Leu721, Glu725, Trp755, Arg766, Phe778, Thr894	Selected steroid-binding pocket residues contributing to progesterone recognition, hydrophobic cavity formation, and polar stabilization.	20.683125	10.343125	61.588875	60 x 60 x 60	0.375	AutoDock 4.2; Lamarckian Genetic Algorithm; 100 docking runs; 25,000,000 energy evaluations; population size 150; mutation rate 0.02; crossover rate 0.8; RMSD clustering tolerance 2.0 Å
GR	4P6X	Asn564, Gln570, Arg611, Phe623, Ile629, Met639, Gln642, Thr739	Selected polar and hydrophobic residues shaping the glucocorticoid-binding pocket and conserved corticosteroid hydrogen-bond network.	6.7235	29.859625	−6.80825	60 x 60 x 60	0.375	AutoDock 4.2; Lamarckian Genetic Algorithm; 100 docking runs; 25,000,000 energy evaluations; population size 150; mutation rate 0.02; crossover rate 0.8; RMSD clustering tolerance 2.0 Å
MR	2AA2	Ser767, Asn770, Gln776, Ser810, Arg817, Met852, Cys942, Thr945	Selected conserved steroid receptor pocket residues involved in mineralocorticoid recognition, polar anchoring, and hydrophobic stabilization.	18.2863	72.3550	19.5303	60 x 60 x 60	0.375	AutoDock 4.2; Lamarckian Genetic Algorithm; 100 docking runs; 25,000,000 energy evaluations; population size 150; mutation rate 0.02; crossover rate 0.8; RMSD clustering tolerance 2.0 Å
