# Supplementary material for: Limitations of Molecular Docking in Predicting the Selectivity of Selective Androgen Receptor Modulators (SARMs): A Comparative Study of YK11 and Ostarine Across Five Nuclear Receptors
Source: Int J Mol Sci. 2026 Jun 26;27(13):5765. doi: 10.3390/ijms27135765 (PMC13361875; doi:10.3390/ijms27135765)
Supplement: Supplementary file 1 [file ijms-27-05765-s001.zip › S2_table.pdf]

Table S2. Complete AutoDock 4.2 docking output values for all receptor–ligand combinations.

Receptor	PDB ID	Ligand	Conformations	RMSD	Binding energy (kcal/mol)	Docking-derived Ki	Intermolecular energy (kcal/mol)	Total internal energy (kcal/mol)	Torsional free energy (kcal/mol)	Unbound energy (kcal/mol)
AR	2AM9	YK11	52	1.97 Å	−13.57	113.14 pM	−14.76	+3.18e+005	+1.19	+3.18e+005
AR	2AM9	Ostarine	2	1.95 Å	−11.08	7.52 nM	−12.87	+2.45e+005	+1.79	+2.45e+005
AR	2AM9	Testosterone	88	1.99 Å	−13.42	145.77 pM	−13.42	+2.45e+005	+0.00	+2.45e+005
ER	1A52	YK11	7	1.58 Å	−11.32	5.02 nM	−12.52	−6.56	+1.19	−6.56
ER	1A52	Ostarine	4	1.59 Å	−10.42	22.99 nM	−12.21	−11.55	+1.79	−11.55
ER	1A52	Estradiol	59	1.57 Å	−10.88	10.67 nM	−10.88	−12.10	+0.00	−12.10
PR	1A28	YK11	47	1.59 Å	−14.03	51.93 pM	−15.22	−15.65	+1.19	−15.65
PR	1A28	Ostarine	8	1.56 Å	−12.05	1.46 nM	−13.84	−15.30	+1.79	−15.30
PR	1A28	Progesterone	52	1.50 Å	−13.04	277.38 pM	−13.34	−14.68	+0.30	−14.68
GR	4P6X	YK11	47	1.52 Å	−14.79	14.32 pM	−15.99	−12.37	+1.19	−12.37
GR	4P6X	Ostarine	1	1.59 Å	−11.60	3.12 nM	−13.39	−14.34	+1.79	−14.34
GR	4P6X	Cortisol	39	1.61 Å	−15.14	7.96 pM	−15.44	−12.17	+0.30	−12.17
MR	2AA2	YK11	69	1.49 Å	−12.28	989.11 pM	−13.48	−9.36	+1.19	−9.36
MR	2AA2	Ostarine	9	1.56 Å	−11.57	3.31 nM	−13.36	−7.93	+1.79	−7.93
MR	2AA2	Aldosterone	66	1.50 Å	−13.55	117.20 pM	−13.85	−9.66	+0.30	−9.66
