# Supplementary material for: Limitations of Molecular Docking in Predicting the Selectivity of Selective Androgen Receptor Modulators (SARMs): A Comparative Study of YK11 and Ostarine Across Five Nuclear Receptors
Source: Int J Mol Sci. 2026 Jun 26;27(13):5765. doi: 10.3390/ijms27135765 (PMC13361875; doi:10.3390/ijms27135765)
Supplement: Supplementary file 1 [file ijms-27-05765-s001.zip › S3_table.pdf]

Table S3. Ligand–receptor interaction fingerprints generated from the best-ranked docking poses.

Ligand	Receptor	Hydrogen-bonding / polar contacts	Other non-covalent contacts	Hydrophobic contacts	Unfavorable contacts
YK11	AR	Conventional H-bonds: Gln711, Trp741, Arg752; C–H bond: Gly708	π-Sigma: Phe764	Alkyl/π-Alkyl: Met742, Met745, Met749, Met787, Val746, Leu873	None reported
YK11	ER	Conventional H-bond: Arg394; C–H bond: Gly521	No π-Sigma or π-Sulfur reported	Alkyl/π-Alkyl: Leu346, Leu349, Leu387, Leu391, Met421, Leu525, His524	None reported
YK11	PR	Conventional H-bonds: Thr894, Val760; C–H bond: Cys891	None specifically reported beyond hydrophobic contacts	Alkyl/π-Alkyl: Leu718, Leu721, Leu763, Phe778	None reported
YK11	GR	Conventional H-bonds: Gln570, Arg611, Gln642, Thr739; C–H bonds: Met639, Asn564	None specifically reported beyond hydrophobic contacts	Alkyl/π-Alkyl: Met560, Met601, Met646, Cys643, Cys736, Leu608, Leu732	None reported
YK11	MR	Conventional H-bond: Asn770	π-Donor H-bond: Ser810; π-Sulfur: Met807	Alkyl/π-Alkyl: Leu766, Leu814, Leu960, Met845, Trp806, Phe941, Ala773	Unfavorable acceptor–acceptor interaction: Phe829
Ostarine	AR	Conventional H-bonds: Gln711, Arg752	Halogen bond: Met745; π-Sigma: Met742	Alkyl/π-Alkyl: Leu701, Leu707, Val746, Met749, Phe764, Met895, Ile899	None reported
Ostarine	ER	Conventional H-bonds: Met421, His524, Arg394	Halogen bonds: Leu346, Glu353, Phe404; π-π T-shaped: Phe404; π-Sigma: Leu525	Alkyl/π-Alkyl: Leu349, Leu387, Leu391, Met388, Ala350, Ile424	None reported
Ostarine	PR	Conventional H-bonds: Asn719, Leu718, Arg766, Gln725; C–H bond: Gly722	Halogen bonds: Leu721, Gly722; π-Sulfur: Met801; π-π stacked: Phe778; π-Sigma: Leu715	Alkyl/π-Alkyl: Leu718, Leu721, Leu763, Met759	None reported
Ostarine	GR	Conventional H-bonds: Arg611, Gln570, Asn564, Cys643; C–H bond: Gly567	Halogen bond: Leu563; π-π T-shaped: Phe623, Phe749; π-Sigma: Met646	Alkyl/π-Alkyl: Met560, Met604, Cys736	Unfavorable donor–donor interaction: Gln642
Ostarine	MR	Conventional H-bonds: Asn770, Thr945, Arg817	Halogen bonds: Phe941, Cys942; π-π T-shaped: Phe829	Alkyl/π-Alkyl: Leu766, Leu769, Leu772, Leu814, Phe956	None reported
Testosterone	AR	Conventional H-bonds: Asn705, Gln711, Arg752	No additional polar/π interactions reported	Alkyl/π-Alkyl: Leu873, Met742, Val746, Met745, Met749, Phe764	None reported
Estradiol	ER	Conventional H-bonds: His524, Arg394	π-π T-shaped: Phe404	Alkyl/π-Alkyl: Leu384, Leu387, Leu391, Met388, Leu525	None reported
Progesterone	PR	Conventional H-bonds: Asn719, Arg766	van der Waals contacts: Val760, Met801, Met759, Phe778, Leu763, Gln725, Leu721, Trp755, Gly722	Alkyl/π-Alkyl: Leu718, Met756, Phe794, Leu715, Cys891, Tyr890, Met759, Met909, Trp755	None reported
Cortisol	GR	Conventional H-bonds: Gln570, Arg611, Leu563, Gln642, Thr739, Asn564	No π-Alkyl or aromatic interactions reported	Alkyl contacts: Met601, Met604, Cys736	None reported

Ligand	Receptor	Hydrogen-bonding / polar contacts	Other non-covalent contacts	Hydrophobic contacts	Unfavorable contacts
Aldosterone	MR	Conventional H-bonds: Asn770, Leu814, Arg817	No π-Alkyl or aromatic interactions reported	Alkyl contacts: Ala773, Cys942, Met807, Met845	None reported
