# Supplementary material for: Limitations of Molecular Docking in Predicting the Selectivity of Selective Androgen Receptor Modulators (SARMs): A Comparative Study of YK11 and Ostarine Across Five Nuclear Receptors
Source: Int J Mol Sci. 2026 Jun 26;27(13):5765. doi: 10.3390/ijms27135765 (PMC13361875; doi:10.3390/ijms27135765)
Supplement: Supplementary file 1 [file ijms-27-05765-s001.zip › S4_table.pdf]

Table S4. Literature-based comparison between AutoDock 4.2-derived inhibition constants and available experimental in vitro binding data for ostarine/enobosarm/MK-2866/S-22.

Ligand	Receptor	AutoDock 4.2-derived Ki	Available experimental binding data	Assay type / receptor system	Source	Interpretation
Ostarine / enobosarm / MK-2866 / S-22	AR / NR3C4	7.52 nM	Ki ≈ 3.8 nM	Competitive radioligand binding; androgen receptor binding assays in SARM medicinal chemistry literature	Mohler et al., J. Med. Chem. 2009, 52, 3597–3617; DOI: 10.1021/jm900280m; PMID: 19432422	Same nanomolar range; partial single-point support for order-of-magnitude AR prediction.
Ostarine / enobosarm / MK-2866 / S-22	ER / ESR1/ESR2	22.99 nM	No peer-reviewed direct Ki, Kd, or IC50 binding value identified	Not available	No primary peer-reviewed direct binding source identified	No numerical experimental comparison possible; computational hypothesis.
Ostarine / enobosarm / MK-2866 / S-22	PR / PGR / NR3C3	1.46 nM	No peer-reviewed direct Ki, Kd, or IC50 binding value identified	Not available	No primary peer-reviewed direct binding source identified	No numerical experimental comparison possible; computational hypothesis.
Ostarine / enobosarm / MK-2866 / S-22	GR / NR3C1	3.12 nM	No peer-reviewed direct Ki, Kd, or IC50 binding value identified	Not available	No primary peer-reviewed direct binding source identified	No numerical experimental comparison possible; computational hypothesis.
Ostarine / enobosarm / MK-2866 / S-22	MR / NR3C2	3.31 nM	No peer-reviewed direct Ki, Kd, or IC50 binding value identified	Not available	No primary peer-reviewed direct binding source identified	No numerical experimental comparison possible; computational hypothesis.
